# Time-restricted eating, liver health, and fecal microbiota in adults with overweight or obesity: A randomized controlled trial

**DOI:** 10.1016/j.jhepr.2026.101956

**Published:** 2026-07-07

**Authors:** Manuel Dote-Montero, Antonio Clavero-Jimeno, Adrián Cortés-Martín, Amaya Lopez-Pascual, Elisa Merchán-Ramírez, Alba Camacho-Cardenosa, Mara Concepción, Maddi Osés, Alejandro López-Vázquez, Francisco J. Amaro-Gahete, Juan J. Martin-Olmedo, Lucas Jurado-Fasoli, Alejandro De-la-O, Patricia V. García Pérez, Julio Gálvez, Alba Rodriguez-Nogales, Federico Garcia, Carmen Mbongo Habimana, Marcos Jiménez Vázquez, Víctor Manuel Alfaro-Magallanes, Matías A. Avila, Jose L. Martín-Rodríguez, Rafael Cabeza, Manuel Muñoz-Torres, Idoia Labayen, Jonatan R. Ruiz

**Affiliations:** 1 Department of Physical Education and Sports, Faculty of Sport Sciences, Sport and Health University Research Institute (iMUDS), University of Granada, Granada, Spain; 2 Obesity and Diabetes Clinical Research Section, Phoenix Epidemiology and Clinical Research Branch, National Institute of Diabetes and Digestive and Kidney Diseases, National Institutes of Health, Phoenix, AZ, USA; 3 Hepatology Laboratory, Solid Tumors Program, CIMA, CCUN, University of Navarra, Pamplona, Spain; 4 Instituto de Investigaciones Sanitarias de Navarra IdiSNA, Pamplona, Spain; 5 Instituto de Investigación Biosanitaria de Granada (ibs.GRANADA), Granada, Spain; 6 Centro de Investigación Biomédica en Red: Fisiopatología de la Obesidad y Nutrición (CIBEROBN), Instituto de Salud Carlos III, Madrid, Spain; 7 Institute for Sustainability & Food Chain Innovation, Department of Health Sciences, Public University of Navarre, Pamplona, Spain; 8 Navarra Institute for Health Research (IdiSNA), Pamplona, Spain; 9 Department of Electrical, Electronic and Communications Engineering, Public University of Navarre, Pamplona, Spain; 10 Department of Physiology, Faculty of Medicine, Sport and Health University Research Institute (iMUDS), University of Granada, Granada, Spain; 11 Radiology Department, University Hospital Clínico San Cecilio, Granada, Spain; 12 Department of Pharmacology, Centro de Investigación Biomédica (CIBM), University of Granada, Granada, Spain; 13 Centro de Investigación Biomédica en Red de Enfermedades Hepáticas y Digestivas (CIBERehd), Instituto de Salud Carlos III, Madrid, Spain; 14 Servicio de Microbiología, Hospital Universitario Clínico San Cecilio, Granada, Spain; 15 Centro de Investigación Biomédica en Red de Enfermedades Infecciosas (CIBERINFEC), Instituto de Salud Carlos III, Madrid, Spain; 16 Radiology Department, Clínica Universidad de Navarra, Pamplona, Spain; 17 LFE Research Group, Department of Health and Human Performance, Faculty of Physical Activity and Sport Sciences, Universidad Politécnica de Madrid, Madrid, Spain; 18 Endocrinology and Nutrition Unit, University Hospital San Cecilio Clinic, Granada, Spain; 19 Department of Medicine, University of Granada, Granada, Spain; 20 CIBER on Frailty and Healthy Aging (CIBERFES), Carlos III Health Institute, Madrid, Spain

**Keywords:** Circadian rhythm, Meal timing, MASLD, Elastography, Hepatokines, Adipokines, Gut-liver axis

## Abstract

**Background & Aims:**

The optimal eating window for time-restricted eating (TRE) remains unclear. We investigated the effects of 8-h TRE combined with usual care (UC, a Mediterranean diet-based education program) *vs.* UC alone over 12 weeks on hepatic fat fraction (HFF), liver health markers, and fecal microbiota in adults with overweight or obesity.

**Methods:**

In this multicenter randomized trial, participants (50% women) were assigned to UC (n = 49), early TRE (n = 49), late TRE (n = 52), or self-selected TRE (n = 47) group. HFF was assessed by magnetic resonance imaging; liver markers included elastography-based parameters, liver enzymes, and circulating biomarkers. Fecal microbiota was analyzed by 16S rRNA gene sequencing.

**Results:**

HFF decreased significantly within the three TRE groups (all *p* ≤0.02). However, there were no between-group differences when comparing early TRE (mean difference [MD] -0.4%, *p* = 0.95), late TRE (MD -1.5%, *p* = 0.15), and self-selected TRE groups (MD -0.7%, *p* = 0.77) with the UC group, or among the TRE groups themselves (all *p* ≥0.41). Similarly, there were no between-group differences in liver health markers and fecal microbiota. Participants with metabolic dysfunction-associated steatotic liver disease at baseline as well as those achieving ≥5% weight loss had greater reductions in HFF than those who did not (MD -2.7 and -2.6%, respectively; both *p* <0.001). A higher proportion of participants in the TRE groups achieved ≥5% weight loss compared with UC (41–44% *vs*. 16%, *p* = 0.001).

**Conclusions:**

These findings suggest that the timing of the eating window in time-restricted eating may not impact HFF or microbiota composition beyond the effects of weight loss; however, the study was not powered for secondary outcomes.

**Impact and implications:**

Time-restricted eating (TRE) is increasingly used for obesity management, but whether the timing of the eating window influences liver health remains unclear. In this 12-week multicenter randomized trial, adding early, late, or self-selected 8-h TRE to Mediterranean diet-based usual care led to within-group reductions in MRI-assessed hepatic fat fraction (HFF); however, it did not confer greater improvements in HFF, liver health markers, or fecal microbiota than usual care alone. Participants with baseline metabolic dysfunction-associated steatotic liver disease (MASLD) and those achieving ≥5% weight loss experienced larger reductions in HFF, suggesting that these reductions in this context are more closely linked to weight loss and baseline steatosis than to eating window timing. Clinically, these findings support prioritizing feasible eating schedules and strategies that help patients attain clinically meaningful weight loss, particularly among individuals with MASLD.

**Clinical Trial registration:**

The study is registered at ClinicalTrials.gov (NCT05310721).

## Highlights

• Early, late, and self-selected 8-h time-restricted eating (TRE) reduced hepatic fat fraction (HFF) within groups over 12 weeks.
• Incorporating TRE into usual care (UC) did not improve liver fat, liver markers, or microbiota *vs.* UC alone.
• Weight loss ≥5% was associated with greater reductions in HFF.
• Participants in the TRE groups were more likely to achieve clinically meaningful weight loss than those receiving UC.
• Eating window timing may be less relevant than weight loss for reducing HFF.

## Graphical abstract

**Figure:**
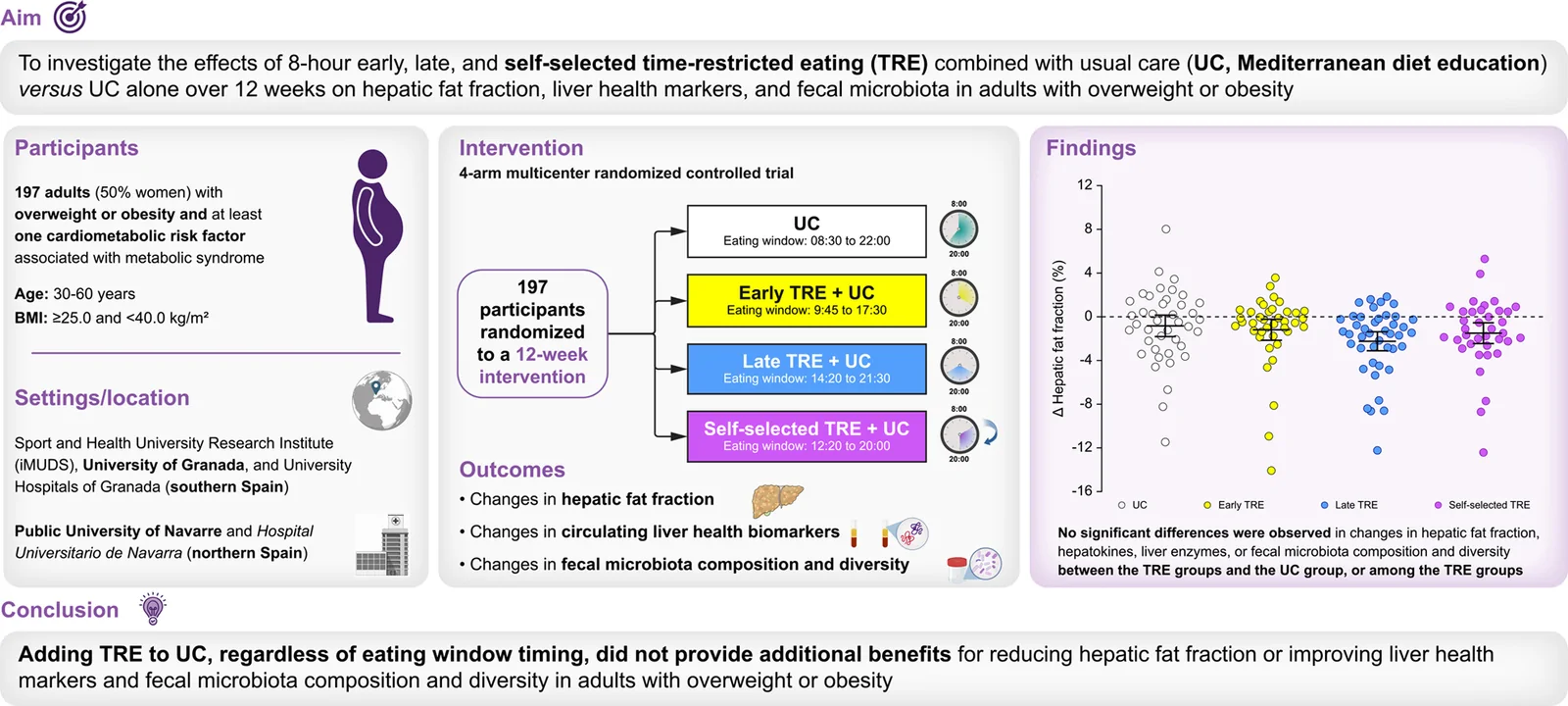

## Introduction

Obesity is a major public health challenge,^1^ increasing the risk of metabolic dysfunction-associated steatotic liver disease (MASLD).^2^ MASLD is characterized by prolonged accumulation of lipids in the liver and is tightly linked to type 2 diabetes and cardiovascular disease,^3^,^4^ thereby placing a substantial burden on healthcare systems.^5^ Although calorie restriction effectively improves hepatic steatosis and metabolic health,[6], [7], [8] its long-term adherence remains challenging.^9^

Time-restricted eating (TRE) has emerged as a promising dietary approach that limits food intake to a predefined eating window of ≤10 h, followed by a fasting period of ≥14 h.[10], [11], [12] Notably, our group^13^ and others have shown that TRE is well tolerated, achieves high adherence rates, and leads to minimal adverse effects, resulting in modest body weight loss and slight improvements of cardiometabolic health in individuals with overweight or obesity.^11^,^12^,^14^ Whether eating window timing influences the efficacy of TRE on hepatic fat fraction (HFF) and liver health markers remains uncertain.^10^

Few studies have investigated the effects of TRE on HFF using magnetic resonance imaging (MRI) or MRI combined with magnetic resonance spectroscopy,[15], [16], [17] both consolidated, non-invasive gold standard methods for measuring HFF. Overall, these studies suggest that TRE reduces HFF while improving liver function. However, limitations such as absence of control group, short duration of the intervention, or exclusive focus on early TRE schedules prevent definitive conclusions. We recently reported that, after a 12-week intervention, early TRE (eating window from ∼09:45 to ∼17:30), late TRE (from ∼14:20 to ∼21:30), and self-selected TRE (from ∼12:20 to ∼20:00) combined with usual care (UC, from ∼08:30 to ∼22:00, a Mediterranean diet-based education program) significantly reduced visceral adipose tissue as measured by MRI, but these reductions did not differ from UC alone.^13^ However, early TRE significantly lowered fasting and nocturnal glucose levels compared with UC and the other TRE schedules, and also decreased subcutaneous abdominal adipose tissue relative to UC.^13^ The liver exhibits robust circadian rhythm, which regulates the expression and activity of enzymes involved in glucose and lipid metabolism peaking in the morning in humans.^18^ Therefore, it is biologically plausible that early TRE elicits a greater impact on HFF and liver health markers.

Additionally, gut-liver axis dysfunction has been identified as a contributing factor to MASLD, with negative alterations in gut microbiota composition and function leading to increased lipid absorption and triggering a proinflammatory cascade that exacerbates hepatic inflammation through dysregulated secretion of hepatokines and cytokines.^19^ Thus, understanding the potential interactions between TRE, gut microbiota composition, and HFF may provide insights into novel therapeutic approaches for MASLD.

In this randomized controlled trial (RCT), we investigated the effects of three distinct TRE schedules over 12 weeks, an 8-h eating window in the early part of the day (early TRE), an 8-h window later in the day (late TRE), and a participant-selected eating window (self-selected TRE), combined with UC, *vs.* UC alone. We focused on changes in HFF measured by MRI, quantitative elastography-based liver markers (including stiffness, attenuation, and viscosity), liver enzymes, circulating liver-related adipokines and hepatokines, and fecal microbiota composition in men and women with overweight or obesity.

## Materials and methods

### Study design

This study is a secondary analysis of a multicenter, parallel-group, investigator-initiated RCT.^20^ The trial received approval from the relevant regulatory bodies and ethics committees in Spain, including the *Servicio Andaluz de Salud*, the *Comité Ético de Investigación Provincial de Granada* (ref. 1/22), and the *Comité Ético de Investigación Clínica de Navarra* (PI_2021/119). The study was registered on ClinicalTrials.gov (identifier: NCT05310721) and conducted in accordance with the Consolidated Standards of Reporting Trials (CONSORT) guidelines for randomized clinical trials. Written informed consent was obtained from all participants before enrollment. The study design is illustrated in Fig. S1.

### Participants and eligibility criteria

White European men and women aged 30–60 years from Spain with overweight or obesity (BMI between 25.0 and 40.0 kg/m^2^) were eligible for inclusion if they also met the following criteria: abdominal obesity (waist circumference ≥95 cm for men and ≥82 cm for women), stable body weight (±3%) for ≥3 months, <150 min/week of moderate-to-vigorous physical activity, a habitual eating window ≥12 h, and at least one cardiometabolic risk factor associated with metabolic syndrome.^20^ Key exclusion criteria included ongoing participation in weight-loss programs, the presence of cardiovascular or chronic diseases (*e.g.* recent myocardial infarction, hemorrhagic stroke, Cushing’s syndrome, adrenocortical insufficiency, or type 1 or 2 diabetes), pregnancy or breastfeeding, and current night-shift work. Further details regarding the eligibility criteria and screening procedures are described elsewhere.^20^

### Study recruitment, enrollment, and randomization

Participants were enrolled in nine consecutives waves of 10–13 individuals per center between 11 April 2022, and 5 December 2022, with final study procedures completed by 6 March 2023. Before enrollment, prospective participants underwent clinical and physical examinations to confirm eligibility. Those who met the inclusion criteria proceeded to baseline assessments.

After baseline assessments, participants were randomized to one of four intervention arms using a stratified, permuted block design. Randomization was stratified by study site and sex, with variable block sizes (4 and 8). Separate randomization lists were generated for each site and stratum. Within each block, participants were randomly assigned in equal proportions to one of the four study groups using a 1:1:1:1 allocation ratio under a parallel-group design.

Personnel responsible for acquiring and analyzing MRI-derived HFF, ultrasound-based outcomes, fasting blood samples, and fecal microbiota composition were blinded to group allocation. In contrast, staff involved in delivering the intervention and performing other assessments were not blinded (open-label design).

### Study assessments and outcomes

#### Hepatic fat fraction

Assessments were conducted at baseline and after the 12-week intervention. We measured HFF using MRI (Siemens 3T MAGNETOM Vida, Siemens Healthineers, Erlangen, Germany) under fasting conditions (minimum 4–5 h). Stages of hepatic steatosis were defined as follows: S0, no steatosis (HFF <5%); S1, mild steatosis (HFF ≥5% and <15%]; S2, moderate steatosis (HFF ≥15% and <25%); and S3, severe steatosis (HFF ≥ 25%).^21^ Participants with HFF ≥5.0% and overweight or obesity were classified as having MASLD, according to current clinical practice guidelines.^3^ MASLD remission was defined as having HFF ≥5.0% at baseline and <5.0% after the 12-week intervention.

#### Adipokines, hepatokines, and adipo-hepatokines

Adipokines, hepatokines, and adipo-hepatokines were quantified using commercial ELISA kits (R&D Systems, Minneapolis, MN, USA), as per the manufacturer’s specifications. The sample type (serum or plasma) and the specific ELISA kits were used for measurements as detailed: adiponectin (DY1065), IL-6 (HS600C), angiopoietin-like 3 (ANGPTL3; DY3829), clusterin (DY5874), sex hormone-binding globulin (SHBG; DY2656), dipeptidyl peptidase IV (DPPIV; DY1180), and retinol-binding protein 4 (RBP4; DY3378) were measured from serum, whereas omentin (DY4254), resistin (DY1359), β-klotho (DY5889), follistatin (DY669), angiopoietin-like 4 (ANGPTL4; DY3485), and growth differentiation factor 15 (GDF15; DY957) were measured from plasma.

#### Fecal microbiota

A total of 182 participants provided fecal samples in sterile containers before and during the last 2 weeks of the intervention for fecal microbiota analysis through 16S rRNA amplicon sequencing. Notably, because of sequencing technical issues and low-quality data, the final analysis included gut microbiota data from 138 participants at both time points of the intervention. These participants were evenly distributed across the different intervention groups (UC: n = 35; early TRE: n = 36; late TRE: n = 36; self-selected TRE: n = 31). Samples were self-collected either the evening before or on the same morning they were delivered to the laboratory. The samples collected the evening before were stored in the home freezer before being transported in cooler containers to the laboratory. Upon arrival, all fecal samples were stored at -80 °C until further analysis. Bacterial DNA was extracted from the samples using the QIAamp PowerFecal Pro DNA Kit (Qiagen, Hilden, Germany) following the manufacturer’s instructions. Library preparation was conducted following the 16S Metagenomic Sequencing Library Preparation Illumina protocol. Furthermore, 16S bacterial rRNA gene was amplified using primers targeting the V3–V4 hypervariable region (341F and 805R primers).

Further details on elastography, liver enzyme, anthropometric, dietary intake, insulin resistance, and fecal microbiota assessments are provided in the Supplementary Methods.

### Study intervention and control condition

Participants assigned to the UC group maintained their habitual eating schedule (≥12-h eating window) and received a healthy lifestyle educational program,^20^ based on the Mediterranean dietary pattern,^22^ and aligned with the World Health Organization’s physical activity recommendations. All participants, including those in the TRE groups, attended twice-monthly sessions led by experienced dietitians as part of this educational program. These meetings addressed six key topics: (1) healthy lifestyle practices based on Mediterranean diet and physical activity guidelines; (2) meal planning and organization; (3) hunger and satiety regulation; (4) interpreting food labels; (5) nutritional myths; and (6) healthy snack selection. Each session concluded with a group discussion of individual challenges, allowing for personalized guidance and peer support. No specific calorie restriction or calorie counting was prescribed during the intervention, and participants in all groups were allowed *ad libitum* food intake.

Participants in the early TRE group selected an 8-h eating window that began no later than 10:00, whereas those in the late TRE group chose an 8-h window starting no earlier than 13:00. The self-selected TRE group established their preferred 8-h eating window before the start of the intervention. All TRE participants were instructed to maintain the same eating window throughout the 12-week intervention and to adhere to it daily (*i.e.* 7 days per week). Consumption of calorie-containing foods or beverages outside the eating window was not permitted; only water, black coffee, and unsweetened tea (free of sugar or artificial sweeteners) were allowed. All participants, including those in the UC group, recorded the timing of their first and last meals each day throughout the intervention using a customized mobile application (EXTREME: com.nnbi.app_extreme; NNBi2020 S L., Navarra, Spain).

Given that Spain is known for having one of the latest dinning hours in Europe—typically around 22:00, compared with 21:00 in Italy, 20:00 in France, 19:00 in Germany, and 18:00 in Sweden—we allowed early TRE participants to choose an 8-h window that started no later than 10:00, enabling a dinner as late as 18:00. Although this may be considered a typical dinner time in other countries, it remains early by Spanish standards.

### Statistical analysis

Sample size and power calculations for the trial were based on the primary outcome (changes in visceral adipose tissue) of the original study, as described elsewhere.^13^ As we focused exclusively on secondary outcomes in this study, separate *a priori* sample size or power estimations were not conducted for these analyses.

The Shapiro-Wilk test was used to assess the normality of data distributions. Data were screened for potential outliers and data entry errors before analysis; however, no observations were excluded, as no physiologically implausible values or technical errors were identified. Owing to skewed distribution, adipokines, hepatokines, and adipo-hepatokines were log_10_-transformed to normalize their distributions for statistical analyses; however, results are presented in the original units to facilitate interpretation. Intervention impact on secondary outcomes (*i.e.* liver parameters, adipokines, hepatokines, adipo-hepatokines, dietary intake, and alpha diversity metrics from fecal microbiota) in response to the 12-week intervention was evaluated using repeated-measures linear mixed-effects models. Changes in individual outcomes were modeled according to intervention group, time, and the group × time interaction, with participant ID included as a random intercept to account for within-participant repeated measurements. Sex was included as a covariate in all models. Analyses were performed under the intention-to-treat principle using restricted maximum likelihood estimation, assuming missing data were missing at random. To adjust for multiple comparisons, Tukey’s method was applied and reported *p* values are Tukey-adjusted. To examine differences in MASLD remission (defined as HFF <5%) across intervention groups among participants with MASLD at baseline, Fisher’s exact test was used. Detailed fecal microbiota and subgroup analyses are provided in the Supplementary Methods.

All statistical analyses were conducted using R (version 4.4.2; R Foundation for Statistical Computing, Vienna, Austria;). A two-sided *p* value <0.05 was considered statistically significant.

## Results

### Baseline characteristics of participants

A total of 197 participants (98 women) were included in the present study (Fig. 1). Table 1 shows baseline descriptive data of the study participants by group. Sample sizes at baseline, post-intervention, and those included in the linear mixed model analyses are reported in Table S1.

**Fig. 1:**
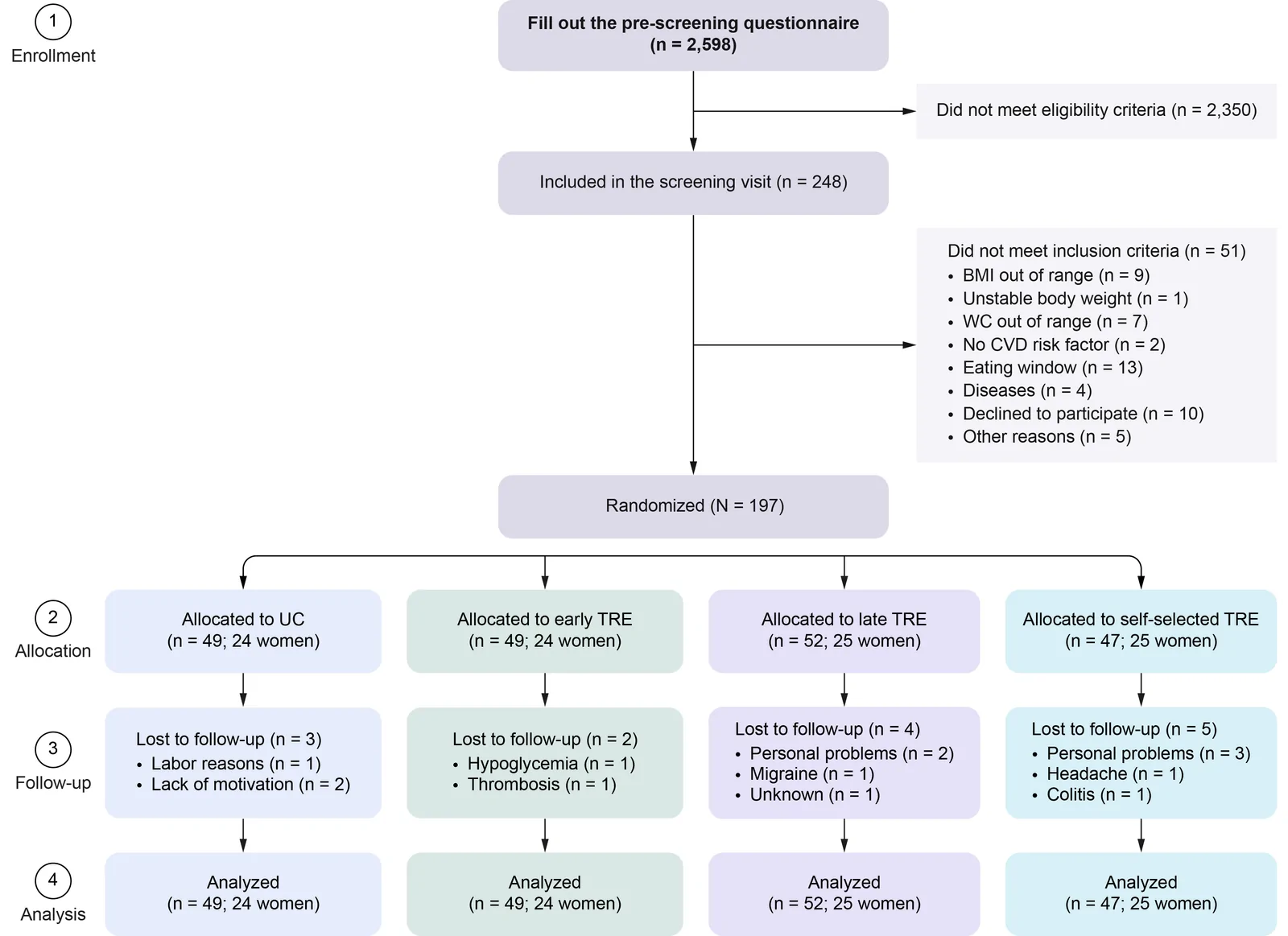
Study flowchart. UC, early TRE, late TRE, or self-selected TRE groups. BMI, body mass index; CVD, cardiovascular disease; TRE, time-restricted eating; UC, usual care; WC, waist circumference. Created in BioRender.

**Table 1:** Baseline characteristics of participants by intervention group.

	UC		Early TRE		Late TRE		Self-selected TRE	
	n		n		n		n	
Age (year)	49	46.7 (6.0)	49	47.2 (6.2)	52	48.0 (6.9)	47	45.2 (5.8)
Women		24 (49.0)		24 (49.0)		25 (48.1)		25 (53.2)
Weight (kg)	49	96.1 (14.4)	49	97.8 (15.3)	52	93.7 (15.4)	47	93.6 (13.8)
Height (cm)	49	169.5 (9.1)	49	169.8 (9.4)	52	169.6 (9.6)	47	169.9 (8.6)
BMI (kg/m^2^)	49	33.4 (3.7)	49	33.8 (3.3)	52	32.4 (3.4)	47	32.4 (3.3)
Waist circumference (cm)	49	102.8 (9.4)	49	103.2 (10.0)	52	101.7 (10.6)	47	99.6 (9.6)
Hip circumference (cm)	49	114.7 (8.9)	49	116.8 (8.4)	52	113.6 (8.0)	47	113.9 (8.2)
Waist-to-hip ratio	49	0.9 (0.1)	49	0.9 (0.1)	52	0.9 (0.1)	47	0.9 (0.1)

**Liver parameters**								
Hepatic fat fraction (%)	47	8.8 (6.7–13.9)	44	6.8 (4.9–10.1)	48	8.9 (6.4–13.4)	43	6.9 (5.3–10.1)
MASLD prevalence∗		43 (91.5)		32 (72.7)		44 (91.7)		35 (81.4)
Stage of hepatic steatosis∗								
S0		4 (8.5)		12 (27.3)		4 (8.3)		8 (18.6)
S1		34 (72.3)		27 (61.4)		35 (72.9)		31 (72.1)
S2		6 (12.8)		4 (9.1)		8 (16.7)		4 (9.3)
S3		3 (6.4)		1 (2.3)		1 (2.1)		0
Liver stiffness (kPa)	38	6.5 (5.9–7.4)	34	6.9 (5.1–7.8)	36	6.7 (5.4–8.3)	31	6.7 (5.6–7.5)
ATI (dB/cm/MHz)	38	0.70 (0.64–0.78)	35	0.69 (0.64–0.76)	36	0.71 (0.67–0.77)	32	0.70 (0.64–0.74)
Viscosity (m/s/kHz)	25	10.7 (9.1–12.3)	23	10.5 (10.1–12.9)	26	10.4 (9.6–12.5)	21	10.3 (9.0–11.8)
ALT (U/L)	49	26 (19–35)	49	23 (17–33)	52	26 (19–34)	47	25 (18–34)
GGT (U/L)	49	30 (21–45)	49	23 (17–31)	52	26 (18–36)	47	23 (17–38)
ALP (U/L)	49	63 (52–75)	49	61 (54–73)	52	66 (56–78)	47	64 (56–78)

**Adipokines**								
Adiponectin (μg/ml)	47	6.1 (4.2–9.1)	47	6.3 (4.3–9.9)	51	5.2 (3.2–7.7)	46	5.8 (4.4–7.5)
IL-6 (pg/ml)	47	2.1 (1.5–2.8)	45	2.1 (1.5–2.6)	49	1.9 (1.5–3.1)	45	1.8 (1.4–2.8)
Omentin (ng/ml)	47	59.5 (44.3–74.6)	45	47.3 (33.6–62.7)	51	49.1 (35.1–61.4)	47	52.2 (40.5–63.2)
Resistin (ng/ml)	47	3.5 (2.8–6.0)	47	3.9 (2.6–5.5)	51	4.0 (2.7–5.5)	47	4.2 (2.8–5.4)

**Hepatokines**								
Angiopoietin-like 3 (ng/ml)	47	424.1 (354.2–528.5)	47	404.6 (314.6–496.3)	50	406.1 (328.7–497.5)	46	383.5 (303.6–450.8)
β-klotho (ng/ml)	47	10.9 (9.8–12.2)	45	10.8 (9.6–11.8)	51	11.0 (9.7–11.9)	47	10.7 (10.2–11.8)
Clusterin (μg/ml)	47	220.9 (192.7–253.7)	47	235.0 (207.0–266.3)	50	220.8 (200.2–262.9)	46	230.8 (193.3–251.1)
Follistatin (ng/ml)	47	0.95 (0.77–1.13)	46	0.97 (0.85–1.16)	49	0.95 (0.73–1.22)	47	0.93 (0.76–1.06)
Sex hormone-binding globulin (nmol/L)	44	17.1 (14.0–31.1)	47	21.3 (10.0–30.6)	49	17.3 (12.9–26.1)	45	21.7 (13.7–24.9)

**Adipo-hepatokines**								
Angiopoietin-like 4 (ng/ml)	47	0.29 (0.17–1.31)	46	0.23 (0.16–0.72)	49	0.22 (0.16–0.68)	47	0.21 (0.15–0.95)
Dipeptidyl peptidase IV (μg/ml)	47	1.01 (0.82–1.24)	47	0.94 (0.79–1.22)	51	0.96 (0.80–1.21)	46	0.95 (0.74–1.08)
Growth differentiation factor 15 (ng/ml)	47	0.28 (0.22–0.35)	47	0.27 (0.24–0.31)	50	0.27 (0.23–0.32)	47	0.25 (0.22–0.28)
Retinol-binding protein 4 (μg/ml)	47	31.7 (22.4–41.8)	47	30.7 (26.2–46.1)	50	35.3 (20.4–46.5)	46	30.7 (22.8–42.8)

**Dietary intake**								
Energy intake (kcal/day)	49	2,059 (1,653–2,306)	49	2,007 (1,741–2,262)	52	1,860 (1,530–2,113)	47	1,882 (1,644–2,294)
Carbohydrate intake (% EI)	49	35 (31–41)	49	35 (32–38)	52	37 (29–40)	47	38 (33–41)
Fat intake (% EI)	49	43 (39–47)	49	45 (40–49)	52	44 (41–48)	47	44 (39–46)
Protein intake (% EI)	49	18 (16–20)	49	17 (15–19)	52	17 (15–19)	47	17 (15–19)
Alcohol intake (% EI)	49	2 (0–5)	49	2 (0–5)	52	0 (0–3)	47	0 (0–3)

**Cardiovascular risk factors**								
Systolic BP (mmHg)	49	125 (13)	48	125 (15)	51	121 (13)	46	123 (16)
Diastolic BP (mmHg)	49	81 (11)	48	82 (11)	51	79 (9)	46	80 (10)
Fasting glucose (mg/dl)	49	93 (88–101)	49	92 (87–98)	52	94 (90–99)	47	91 (87–98)
Fasting insulin (mU/L)	49	11.6 (8.7–13.5)	49	10.1 (7.8–12.7)	50	10.4 (8.4–14.2)	45	9.0 (7.3–13.9)
HOMA-IR	49	2.68 (2.20–3.40)	49	2.36 (1.91–3.19)	50	2.44 (1.90–3.42)	45	2.00 (1.72–3.10)
HbA1c (%)	49	5.3 (5.2–5.6)	49	5.3 (5.1–5.5)	52	5.4 (5.2–5.5)	47	5.3 (5.1–5.5)
Total cholesterol (mg/dl)	49	211 (184–226)	49	205 (186–226)	52	210 (188–252)	47	208 (184–232)
HDL-C (mg/dl)	49	56 (45–64)	49	51 (45–58)	52	55 (46–64)	47	52 (46–60)
LDL-C (mg/dl)	49	128 (107–149)	49	126 (119–147)	52	138 (115–160)	47	129 (114–154)
Triglycerides (mg/dl)	49	122 (84–158)	49	114 (84–165)	52	112 (88–150)	47	114 (89–156)

Data are presented as mean (SD) when normally distributed, median (first quartile–third quartile) when not normally distributed, or as n (%).

ALP, alkaline phosphatase; ALT, alanine aminotransferase; ATI, attenuation imaging coefficient; BP, blood pressure; EI, energy intake; GGT, gamma-glutamyl transferase; HbA1c, glycated hemoglobin; HDL-C, high-density lipoprotein cholesterol; HOMA-IR, homeostasis model assessment of insulin resistance; LDL-C, low-density lipoprotein cholesterol; TRE, time-restricted eating; UC, usual care.

∗ Calculated based on the maximum number of participants with available hepatic fat fraction data. Metabolic dysfunction-associated steatotic liver disease (MASLD) prevalence was defined as the number and percentage of participants with hepatic fat fraction (HFF) ≥5%. Stages of hepatic steatosis were defined as follows: S0, no steatosis (HFF<5%); S1, mild steatosis (HFF ≥5% and <15%); S2, moderate steatosis (HFF ≥15% and <25%); and S3, severe steatosis (HFF ≥25%).^21^.

### Hepatic fat fraction, liver stiffness, attenuation, viscosity, and liver enzymes

We observed significant reductions in HFF within the early TRE (mean change -1.2%, 95% CI -2.2 to -0.2, *p* = 0.02), late TRE (mean change -2.3%, 95% CI -3.3 to -1.3, *p* <0.001), and self-selected TRE groups (mean change -1.5%, 95% CI -2.6 to -0.5, *p* <0.001), but not in the UC group (mean change -0.8%, 95% CI -1.8 to 0.2, *p* = 0.12) (Table S1). However, no statistically significant differences were detected in HFF changes between the early TRE (mean difference -0.4%, 95% CI -2.3 to 1.5, *p* = 0.95), late TRE (mean difference -1.5%, 95% CI -3.3 to 0.3; *p* = 0.15), and self-selected TRE groups (mean difference -0.7%, 95% CI -2.6 to 1.2, *p* = 0.77) compared with the UC group (Fig. 2 and Table 2). No significant differences were observed across TRE groups (Fig. 2 and Table 3). Men had a higher prevalence of MASLD than women (n = 88 *vs*. n = 66, *p* <0.001). The percentage of participants with resolution of MASLD at 12 weeks was similar across all groups (UC 4.7% [n = 2], early TRE 15.6% [n = 5], late TRE 6.8% [n = 3], self-selected TRE 20.0% [n = 7]; *p* = 0.11).

**Fig. 2:**
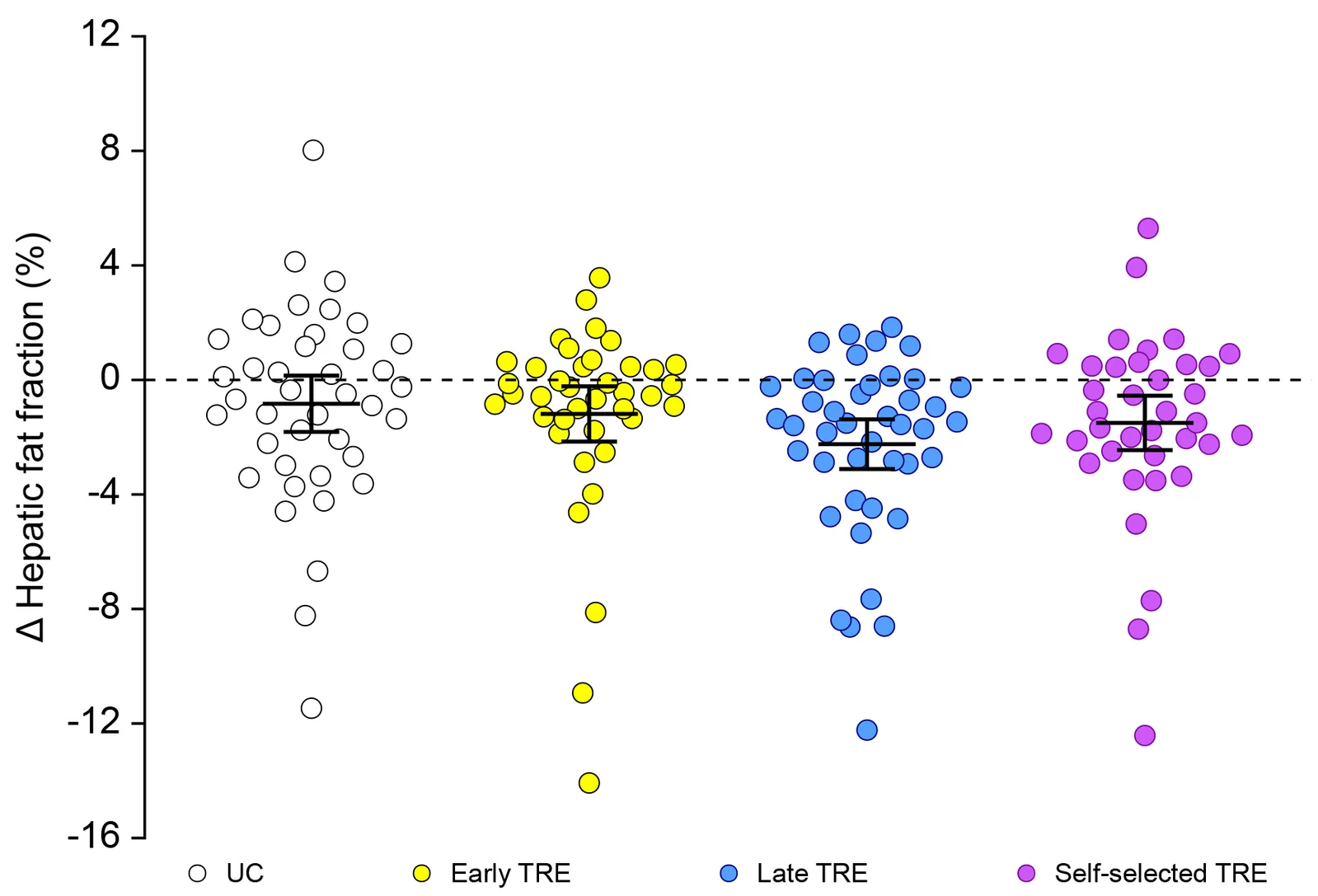
Changes in hepatic fat fraction among the UC, early TRE, late TRE, and self-selected TRE groups after the 12-week intervention. Data are raw means with 95% confidence intervals (CIs). Between-group differences in changes were evaluated using repeated-measures linear mixed-effects models including intervention group, time, and the group × time interaction, with participant ID as a random intercept and sex as a covariate; post hoc comparisons were Tukey-adjusted. No statistically significant differences in changes in hepatic fat fraction were detected across all groups after the intervention. TRE, time-restricted eating; UC, usual care.

**Table 2:** Changes in liver parameters, adipokines, hepatokines, adipo-hepatokines, and dietary intake endpoints in the TRE groups compared with the UC group after the 12-week intervention.

Endpoint	Early TRE *vs.* UC	Late TRE *vs*. UC	Self-selected TRE *vs*. UC
**Liver parameters**			
Hepatic fat fraction (%)	-0.4 (-2.3 to 1.5)	-1.5 (-3.3 to 0.3)	-0.7 (-2.6 to 1.2)
Liver stiffness (kPa)	0.4 (-1.1 to 1.9)	0.6 (-0.9 to 2.1)	0.1 (-1.5 to 1.6)
ATI (dB/cm/MHz)	-0.06 (-0.11 to 0.00)	-0.04 (-0.09 to 0.02)	-0.03 (-0.09 to 0.03)
Viscosity (m/s/kHz)	-0.6 (-3.4 to 2.1)	0.5 (-2.1 to 3.2)	0.4 (-2.4 to 3.3)
ALT (U/L)	-1 (-8 to 6)	-3 (-10 to 4)	-3 (-10 to 4)
GGT (U/L)	1 (-11 to 13)	0 (-11 to 12)	0 (-12 to 12)
ALP (U/L)	3 (-3 to 8)	-1 (-7 to 4)	0 (-5 to 5)

**Adipokines**			
Adiponectin (μg/ml)	-0.5 (-2.9 to 1.9)	0.3 (-2.1 to 2.7)	0.2 (-2.3 to 2.7)
IL-6 (pg/ml)	-0.3 (-1.3 to 0.7)	-0.5 (-1.6 to 0.5)	-0.3 (-1.3 to 0.8)
Omentin (ng/ml)	0.6 (-14.3 to 15.4)	8.7 (-5.9 to 23.2)	2.5 (-12.4 to 17.4)
Resistin (ng/ml)	0.8 (-0.8 to 2.3)	0.0 (-1.5 to 1.5)	0.0 (-1.5 to 1.6)

**Hepatokines**			
Angiopoietin-like 3 (ng/ml)	27.7 (-57.5 to 112.9)	42.9 (-41.6 to 127.4)	42.6 (-43.9 to 129.1)
β-klotho (ng/ml)	0.8 (-1.4 to 2.9)	0.8 (-1.3 to 3.0)	1.6 (-0.6 to 3.8)
Clusterin (μg/ml)	-1.4 (-43.1 to 40.2)	-1.5 (-42.8 to 39.7)	-4.3 (-46.6 to 37.9)
Follistatin (ng/ml)	-0.02 (-0.18 to 0.15)	0.00 (-0.16 to 0.16)	0.01 (-0.15 to 0.18)
Sex hormone-binding globulin (nmol/L)	7.1 (-7.0 to 21.1)	-0.3 (-14.1 to 13.5)	6.8 (-7.4 to 21.0)

**Adipo-hepatokines**			
Angiopoietin-like 4 (ng/ml)	0.23 (-0.20 to 0.67)	0.27 (-0.16 to 0.70)	0.16 (-0.28 to 0.60)
Dipeptidyl peptidase IV (μg/ml)	-0.04 (-0.21 to 0.14)	0.06 (-0.11 to 0.23)	0.05 (-0.12 to 0.23)
Growth differentiation factor 15 (ng/ml)	-0.01 (-0.04 to 0.02)	-0.00 (-0.04 to 0.03)	0.01 (-0.02 to 0.04)
Retinol-binding protein 4 (μg/ml)	-1.0 (-9.8 to 7.7)	-1.2 (-9.9 to 7.5)	0.0 (-8.9 to 8.9)

**Dietary intake**			
Energy intake (kcal/day)	-304 (-565 to -44)∗	-145 (-404 to 115)	-243 (-510 to 24)
Carbohydrate intake (% EI)	1 (-3 to 5)	0 (-4 to 4)	0 (-5 to 4)
Fat intake (% EI)	-1 (-5 to 4)	-1 (-6 to 3)	-1 (-6 to 4)
Protein intake (% EI)	0 (-2 to 2)	0 (-2 to 3)	1 (-1 to 3)
Alcohol intake (% EI)	0 (-2 to 1)	1 (-1 to 2)	0 (-1 to 2)

Data are presented as estimated mean differences and 95% confidence intervals (CIs) between groups and were calculated by first computing the difference between the post-intervention and the pre-intervention values within each group. Then, differences between the groups were computed as early TRE minus UC, late TRE minus UC, and self-selected TRE minus UC. Between-group differences in changes were evaluated using repeated-measures linear mixed-effects models including intervention group, time, and the group × time interaction, with participant ID as a random intercept and sex as a covariate. Post hoc comparisons were Tukey-adjusted.

ALP, alkaline phosphatase; ALT, alanine aminotransferase; ATI, attenuation imaging coefficient; EI, energy intake; GGT, gamma-glutamyltransferase; TRE, time-restricted eating; UC, usual care.

∗ Significant differences between each TRE group and the UC group as determined by *post hoc* Tukey correction for multiple comparisons (*p* <0.05).

**Table 3:** Changes in liver parameters, adipokines, hepatokines, adipo-hepatokines, and dietary intake endpoints in the TRE groups compared with each other after the 12-week intervention.

Endpoint	Early TRE *vs*. late TRE	Early TRE *vs*. self-selected TRE	Late TRE *vs*. self-selected TRE
**Liver parameters**			
Hepatic fat fractin (%)	1.1 (-0.7 to 3.0)	0.3 (-1.6 to 2.2)	-0.8 (-2.7 to 1.1)
Liver stiffness (kPa)	-0.2 (-1.7 to 1.3)	0.3 (-1.3 to 1.9)	0.5 (-1.1 to 2.1)
ATI (dB/cm/MHz)	-0.02 (-0.08 to 0.04)	-0.02 (-0.08 to 0.03)	0.00 (-0.06 to 0.06)
Viscosity (m/s/kHz)	-1.1 (-3.8 to 1.6)	-1.0 (-3.9 to 1.8)	0.1 (-2.7 to 2.9)
ALT (U/L)	2 (-5 to 9)	2 (-5 to 9)	0 (-7 to 7)
GGT (U/L)	1 (-11 to 12)	1 (-11 to 13)	0 (-12 to 12)
ALP (U/L)	4 (-1 to 9)	3 (-3 to 8)	-1 (-7 to 4)

**Adipokines**			
Adiponectin (μg/ml)	-0.8 (-3.2 to 1.6)	-0.7 (-3.1 to 1.7)	0.1 (-2.3 to 2.5)
IL-6 (pg/ml)	0.2 (-0.8 to 1.3)	0.0 (-1.1 to 1.0)	-0.3 (-1.3 to 0.8)
Omentin (ng/ml)	-8.1 (-22.5 to 6.3)	-1.9 (-16.6 to 12.8)	6.2 (-8.2 to 20.6)
Resistin (ng/ml)	0.8 (-0.7 to 2.3)	0.7 (-0.8 to 2.2)	-0.1 (-1.6 to 1.4)

**Hepatokines**			
Angiopoietin-like 3 (ng/ml)	-15.2 (-98.4 to 68.0)	-14.8 (-100.1 to 70.4)	0.3 (-84.2 to 84.8)
β-klotho (ng/ml)	-0.1 (-2.2 to 2.0)	-0.8 (-3.0 to 1.3)	-0.7 (-2.9 to 1.4)
Clusterin (μg/ml)	0.1 (-40.6 to 40.9)	2.9 (-38.8 to 44.7)	2.8 (-38.5 to 44.1)
Follistatin (ng/ml)	-0.02 (-0.18 to 0.14)	-0.03 (-0.19 to 0.13)	-0.01 (-0.17 to 0.15)
Sex hormone-binding globulin (nmol/L)	7.4 (-6.3 to 21.0)	0.3 (-13.7 to 14.3)	-7.1 (-20.9 to 6.7)

**Adipo-hepatokines**			
Angiopoietin-like 4 (ng/ml)	-0.04 (-0.46 to 0.38)	0.08 (-0.36 to 0.51)	0.11 (-0.31 to 0.54)
Dipeptidyl peptidase IV (μg/ml)	-0.09 (-0.26 to 0.07)	-0.09 (-0.26 to 0.08)	0.01 (-0.17 to 0.18)
Growth differentiation factor 15 (ng/ml)	-0.01 (-0.04 to 0.02)	-0.02 (-0.06 to 0.01)	-0.02 (-0.05 to 0.02)
Retinol-binding protein 4 (μg/ml)	0.2 (-8.4 to 8.8)	-1.0 (-9.8 to 7.8)	-1.2 (-9.9 to 7.5)

**Dietary intake**			
Energy intake (kcal/day)	-160 (-417 to 98)	-61 (-326 to 203)	98 (-165 to 362)
Carbohydrate intake (% EI)	1 (-3 to 5)	1 (-3 to 6)	1 (-4 to 5)
Fat intake (% EI)	0 (-4 to 5)	0 (-4 to 5)	0 (-5 to 4)
Protein intake (% EI)	0 (-3 to 2)	-1 (-3 to 1)	0 (-3 to 2)
Alcohol intake (% EI)	-1 (-3 to 1)	-1 (-3 to 1)	0 (-2 to 2)

No statistically significant differences were detected in liver parameters, adipokines, hepatokines, adipo-hepatokines, or dietary intake across the TRE groups after the intervention. Data are presented as estimated mean differences and 95% confidence intervals (CIs) between groups and were calculated by first computing the difference between the post-intervention and the pre-intervention values within each group. Then, differences between the groups were computed as early TRE minus late TRE, early TRE minus self-selected TRE, and late TRE minus self-selected TRE. Between-group differences in changes were evaluated using repeated-measures linear mixed-effects models including intervention group, time, and the group × time interaction, with participant ID as a random intercept and sex as a covariate; post hoc comparisons were Tukey-adjusted.ALP, alkaline phosphatase; ALT, alanine aminotransferase; ATI, attenuation imaging coefficient; EI; energy intake; GGT, gamma-glutamyltransferase; TRE, time-restricted eating.

Interestingly, in exploratory analyses, when participants in the TRE groups were categorized according to the presence of MASLD at baseline (≥5.0% HFF), we observed a significantly greater reduction in HFF in those with MASLD than in those without the condition (mean difference -2.7%, 95% CI -4.1 to -1.2, *p* <0.001; Fig. S6).

### Adipokines, hepatokines, adipo-hepatokines, and correlations with changes in hepatic fat fraction

There were no statistically significant differences in changes in adipokines, hepatokines, and adipo-hepatokines between any TRE group and the UC group (all *p* ≥0.13; Fig. 3 and Table 2), nor among the TRE groups (all *p* ≥0.24; Fig. 3 and Table 3). Reductions in HFF over time were significantly associated with decreases in several hepatokines (*i.e.* ANGPTL3, β-klotho, clusterin, and follistatin) (all r ≥0.263, all *p* = 0.001; Table S2), and with reductions in selected adipo-hepatokines, specifically DPPIV and RBP4 (both r ≥0.307, both *p* <0.001; Table S2) across all samples, and in TRE groups alone (all r ≥0.222, all *p* ≤0.018; Table S2).

**Fig. 3:**
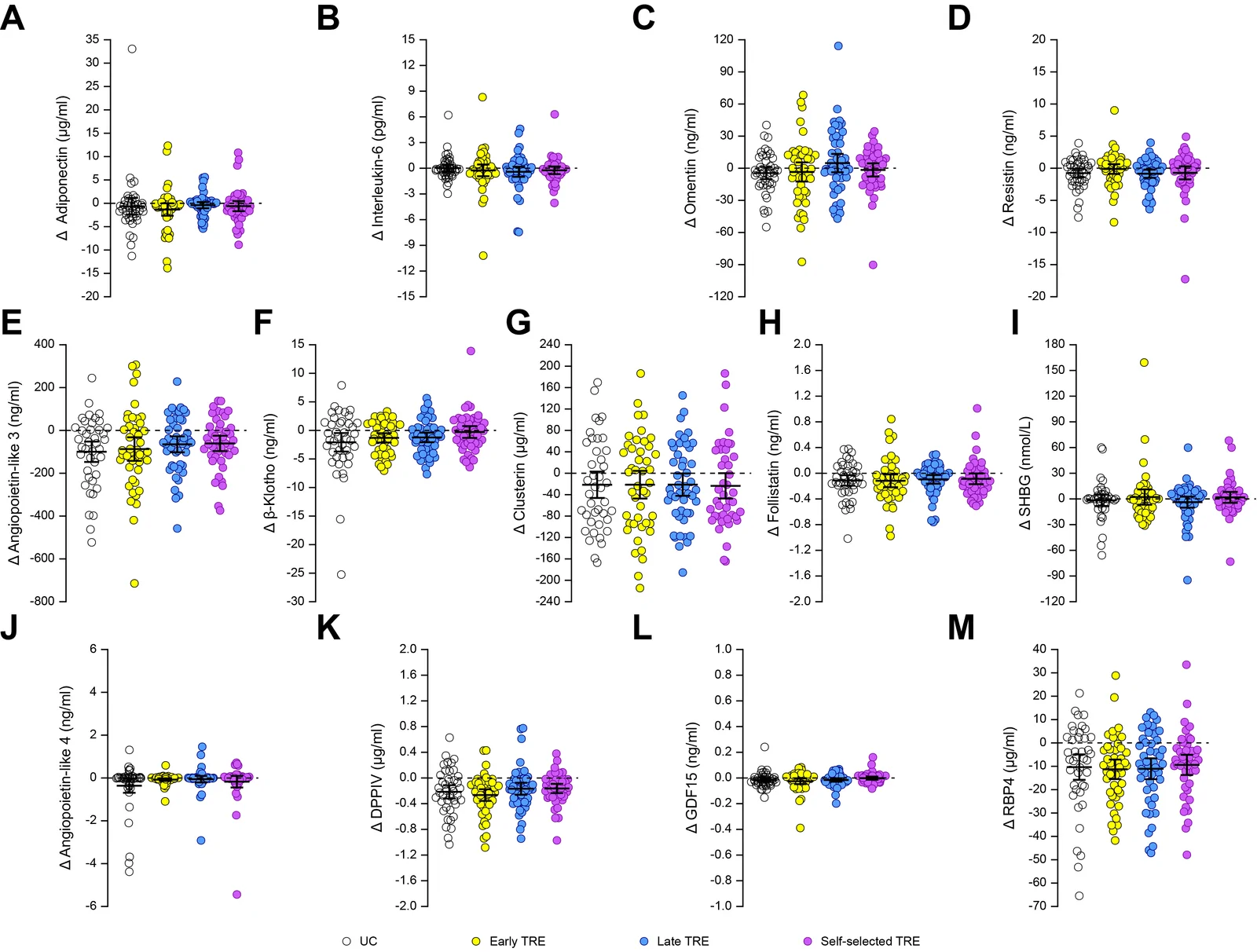
Changes in circulating adipokines, hepatokines, and adipo-hepatokines after the 12-week intervention. (A) Adiponectin, (B) IL-6, (C) omentin, (D) resistin, (E) ANGPTL3, (F) β-klotho, (G) clusterin, (H) follistatin, (I) SHBG, (J) ANGPTL4, (K) DPPIV, (L) GDF15, and (M) RBP4 among the UC, early TRE, late TRE, and self-selected TRE groups after the 12-week intervention. Data are presented as raw means with 95% confidence intervals (CIs). Between-group differences in changes were evaluated using repeated-measures linear mixed-effects models including intervention group, time, and the group × time interaction, with participant ID as a random intercept and sex as a covariate; post hoc comparisons were Tukey-adjusted. No statistically significant differences were detected in changes in the adipokines, hepatokines, or adipo-hepatokines across the groups after the intervention. ANGPTL3, angiopoietin-like 3; ANGPTL4, angiopoietin-like 4; CI, confidence interval; DPPIV, dipeptidyl peptidase IV; GDF15, growth differentiation factor 15; IL-6, interleukin-6; RBP4, retinol-binding protein 4; SHBG, sex hormone-binding globulin; TRE, time-restricted eating; UC, usual care.

### Fecal microbiota composition

Alpha and beta diversity indices of fecal microbiota before and after the intervention are shown in Table S3 and Fig. S3A–D, respectively. Statistically significant differences were observed neither in baseline fecal microbiota composition (*p* = 0.29) nor in beta (*p* = 0.30) and alpha (all *p* ≥0.42) diversity indices across all groups (Fig. S2). There were no statistically significant differences between groups in changes in alpha and beta diversity indices (all *p* ≥0.16; Fig. 4A–E). The UC group showed the highest number of taxa that changed during the 3-month intervention (Fig. S4A). No statistically significant differences were identified when evaluating specific changes in fecal microbiota composition between groups through Microbiome Multivariable Associations with Linear Models (MaAsLin 3) analysis after false discovery rate (FDR) adjustment (all *p* ≥0.60; Fig. 4F, G and Fig. S3E). Fig. S5 shows within-group linear discriminant analysis effect size (LEfSe) analyses for metabolic pathway biomarker discovery. MaAsLin 3 analysis revealed no significant between-group differences in changes in bacterial functionality during the intervention after FDR adjustment (all *p* ≥0.99).

**Fig. 4:**
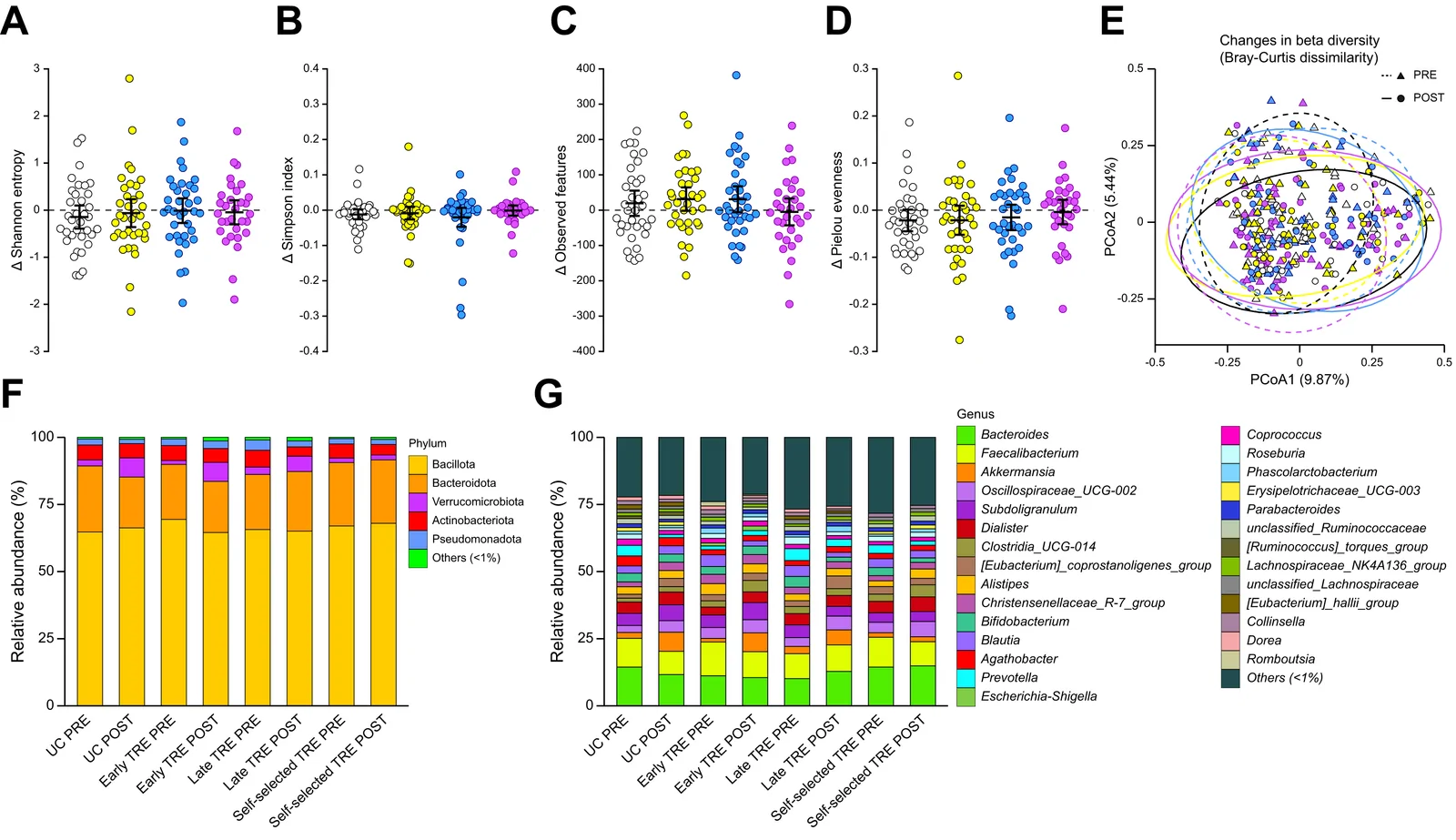
Changes in diversity and microbial taxonomic composition among the UC, early TRE, late TRE, and self-selected TRE groups after the 12-week intervention. (A–E) No statistically significant differences were detected in changes in alpha and beta diversity indices across all groups after the intervention. Changes in alpha diversity (A-D) were evaluated using repeated-measures linear mixed-effects models including intervention group, time, and the group × time interaction, with participant ID as a random intercept and sex as a covariate; post-hoc comparisons were Tukey-adjusted. Changes in beta diversity (E) were assessed using PERMANOVA, including assigned group, assessment time, and their interaction terms. (F and G) Microbial taxonomic composition at the (F) phylum and (G) genus level in fecal samples for the pre-intervention (PRE) and post-intervention (POST) time points for each intervention group. The bars show the mean proportion of relative abundance. The “Others” category in the graphs represents the aggrupation of bacterial groups with less than 1% relative abundance. Changes in bacterial composition were evaluated using MaAsLin3 algorithm, including assigned group, assessment time, and their interaction terms, with sex as a covariate. No statistically significant differences were detected after applying Benjamini-Hochberg false discovery rate (FDR). PCoA, principal coordinates analysis; TRE, time-restricted eating; UC, usual care.

### Association of clinically meaningful weight loss with changes in all study outcomes

Descriptive characteristics of participants who lost <5% or ≥5% of their initial body weight are presented in Table S4. The proportion of participants who achieved clinically meaningful body weight loss (≥5%; so-called responders) significantly differed among the groups (UC, 16%; early TRE, 41%; late TRE, 44%; self-selected TRE, 43%; *p* = 0.001; Fig. S7). Specifically, those who achieved a reduction in body weight of at least 5% showed a greater decrease in HFF (mean difference -2.6%, 95% CI -3.5 to -1.6, *p* <0.001; Fig. S8 and Table S5) and alanine aminotransferase (ALT; mean difference -9 mg/dl, 95% CI -13 to -5, *p* <0.001; Table S5). However, no other significant differences were observed in changes in liver stiffness, attenuation imaging coefficient (ATI), or other liver enzyme levels (all *p* ≥0.21; Table S5). The percentage of participants with resolution of MASLD at 12 weeks was similar in both groups (<5% body weight loss: 7.5% [n = 7]; ≥5% body weight loss: 18.9% [n = 10]; *p* = 0.09). Moreover, participants losing ≥5% of their initial body weight had a statistically significant reduction in adipokine omentin (mean difference -9.8 ng/ml; 95% CI -17.8 to -1.7, *p* = 0.046; Fig. S9C and Table S5), and the hepatokines ANGPTL3 (mean difference -48.8 ng/ml, 95% CI -96.1 to -1.5, *p* = 0.046; Fig. S9E and Table S5) and clusterin (mean difference -32.5 μg/ml, 95% CI -55.4 to -9.6, *p* = 0.02; Fig. S9G and Table S5).

Results on dietary intake, correlations between changes in HFF, fecal microbiota, and insulin resistance, eating window timing and adherence, and intervention effects by sex are provided in the Supplementary Methods.

## Discussion

This secondary analysis of RCT suggests that adding TRE, regardless of the timing of the eating window, to a Mediterranean diet-based nutritional education program (UC) for 12 weeks did not offer any additional benefits over UC alone for reducing HFF, improving liver health markers, or modulating fecal microbiota composition in adults with overweight or obesity. Notably, participants from TRE groups with MASLD at baseline showed larger reductions in HFF than those without MASLD, indicating that TRE could be more effective in individuals with pre-existing hepatic steatosis, consistent with recent studies.^23^ In exploratory analyses, we observed that participants who achieved clinically significant weight loss (≥5%), 91% of whom were from the TRE groups, exhibited greater reductions in HFF, along with improvements in ALT, the adipokine omentin, and the hepatokines ANGPTL3 and clusterin, compared with those who did not. These findings suggest that improvements in HFF and liver health markers are more closely related to the extent of weight loss than to the eating window timing.

Our results align with those of previous trials showing that TRE can improve hepatic fat-related outcomes, but these effects do not appear to differ substantially according to eating window timing.[15], [16], [17],^24^ Prior studies comparing early *vs.* late TRE,^15^ TRE plus calorie restriction *vs.* calorie restriction alone,^16^ or different TRE schedules combined with hypocaloric diets^24^ have generally reported similar improvements in hepatic fat, steatosis, or liver health markers across groups. This pattern aligns with our finding that HFF decreased within the TRE groups but did not differ significantly between TRE schedules or compared with UC. These findings suggest that the observed liver-related improvements are more likely explained by energy deficit, possibly induced by the shortened eating window, than by the specific timing of food intake. Nevertheless, we observed slightly higher MASLD remission rates in the early and self-selected TRE groups than in the UC and late TRE groups; however, these differences did not reach statistical significance. Potential explanations include the alignment of early or self-selected eating windows with circadian rhythms, which facilitate improved metabolic regulation. Indeed, we previously reported in this cohort that early TRE reduced fasting and nocturnal glucose levels compared with UC, late TRE, and self-selected TRE groups.^13^ Recent evidence suggests that MASLD is characterized by altered circadian metabolic regulation, including nighttime impairments in hepatic and peripheral insulin sensitivity, increased endogenous glucose production, and altered lipid handling.^25^ In this context, early TRE may be particularly relevant by reducing evening energy intake and improving nocturnal glycemic homeostasis.

Notably, participants in the TRE groups with pre-existing MASLD at baseline experienced a significantly greater reduction in HFF than those without the condition (-2.1% *vs*. +0.5%). This finding suggests that individuals with elevated baseline hepatic fat derive greater hepatic benefit from TRE, potentially owing to a larger capacity for improvement. The association between reductions in HFF and homeostasis model assessment of insulin resistance (HOMA-IR) further supports the close link between hepatic steatosis and metabolic dysfunction in MASLD.^26^ Overall, these results indicate that TRE may be particularly relevant for individuals with early hepatic steatosis.

A higher proportion of participants in the TRE groups achieved clinically meaningful body weight loss (≥5%) compared with UC group. In exploratory analyses, ≥5% weight loss was associated with greater reductions in HFF (-3.1% *vs*. -0.5%), ALT, and several circulating biomarkers, including ANGPTL3, clusterin, and omentin. These findings suggest that the degree of weight loss is a key driver of hepatic fat reduction and related biomarker changes, thereby reflecting a healthier hepatokine profile.^27^ However, MASLD remission rates did not differ between responders and non-responders, and these effects were not evident in the direct comparisons between TRE and UC. Thus, TRE may primarily improve liver-related outcomes by facilitating energy deficit and weight loss, rather than through the specific timing of the eating window. Future trials with mechanistic endpoints are needed to determine whether additional pathways, such as improved hepatic insulin sensitivity, reduced inflammation, altered lipid metabolism, or gut-liver axis modulation, contribute independently of weight loss.

One proposed mechanism underlying the metabolic benefits of TRE is modulation of the gut microbiota.^28^ However, current evidence remains inconsistent. In our study, TRE did not significantly alter fecal microbiota composition, alpha diversity, or beta diversity compared with UC, and changes in HFF were not associated with microbiota changes. These findings suggest that the hepatic effects observed in our trial were unlikely to be primarily mediated by gut microbiota alterations. Our results are consistent with those of previous TRE studies in adults with obesity,[29], [30], [31] which have generally reported no major effects on microbiome composition or diversity after short-to medium-term interventions. Importantly, our study extends this evidence through a larger, sex-balanced, controlled design comparing different TRE schedules. Future studies should determine whether microbiome responses to TRE depend on eating window timing, sample collection timing, time since last meal, dietary composition, or sequencing methodology.

This study presents several limitations. It is a secondary analysis, and therefore the power calculation of sample size was not based on HFF, other liver health markers, or fecal microbiota composition, but on visceral adipose tissue changes,^13^ the main outcome of the study. Participants had normal or only mildly elevated HFF, liver stiffness, liver enzymes, and cardiometabolic risk factors at baseline, which may have reduced the potential to detect larger effects compared with trials that enrolled participants with MASLD or more advanced metabolic disease. Moreover, the 12-week intervention period may not be long enough to observe substantial differences between intervention groups. Furthermore, although the methodology used for microbiota analyses was robust, reliance on 16S rRNA profiling rather than shotgun metagenomics may have limited the detection of functional microbial shifts. The absence of a non-intervention control group also restricts interpretation of hepatic fat, liver health, and fecal microbiota outcomes. Finally, emerging evidence suggests that fecal microbiota composition is influenced by circadian rhythms and that the timing of stool collection affects replicability.^32^ In addition, the findings may not be generalizable beyond the Spanish population because of cultural differences in meal timing, as Spain is characterized by a later meal schedule compared with other countries.

## Conclusions

In this secondary analysis of RCT, which implemented three different TRE schedules, the findings suggest that adding TRE to a Mediterranean diet-based nutritional program does not confer additional benefits for reducing HFF, improving liver health markers, or modulating fecal microbiota composition beyond those achieved with dietary education alone in participants with overweight or obesity. Our exploratory analysis suggests that TRE is a highly promising and targeted strategy for managing early stages of MASLD, given its demonstrated ability to induce significant reductions in HFF in individuals with pre-existing hepatic steatosis. Importantly, participants who achieved a clinically meaningful weight loss (≥5%)—the majority of whom belonged to the TRE groups (91%)—exhibited greater reductions in HFF, along with improvements in ALT, omentin, ANGPTL3, and clusterin levels, compared with those who did not. These preliminary findings suggest that liver-related improvements may be more closely linked to the degree of weight loss than to eating window timing, with TRE potentially contributing by facilitating weight reduction. Further adequately powered trials are warranted to corroborate our results and determine whether the timing of the eating window, particularly early *vs.* late TRE, differentially impacts HFF, liver health markers, and underlying mechanisms, with a focus on the gut-liver axis. Moreover, further research is needed in those with MASLD, cirrhosis, and chronic diseases, where circadian factors may play a key role.

## Abbreviations

ALP, alkaline phosphatase; ALT, alanine aminotransferase; ANGPTL3, angiopoietin-like 3; ANGPTL4, angiopoietin-like 4; ATI, attenuation imaging coefficient; BP, blood pressure; CONSORT, Consolidated Standards of Reporting Trials; DPPIV, dipeptidyl peptidase IV; EI, energy intake; FDR, false discovery rate; GDF15, growth differentiation factor 15; GGT, gamma-glutamyl transferase; HbA1c, glycated hemoglobin; HDL-C, high-density lipoprotein cholesterol; HFF, hepatic fat fraction; HOMA-IR, homeostasis model assessment of insulin resistance; LDL-C, low-density lipoprotein cholesterol; LEfSe, linear discriminant analysis effect size; MaAsLin 3, Microbiome Multivariable Associations with Linear Models; MASLD, metabolic dysfunction-associated steatotic liver disease; MRI, magnetic resonance imaging; NCBI, National Center for Biotechnology Information; PCoA, principal coordinates analysis; RBP4, retinol-binding protein 4; RCT, randomized controlled trial; SHBG, sex hormone-binding globulin; TRE, time-restricted eating; UC, usual care.

## Authors’ contributions

Conceptualization: MDM, IL, JRR. Methodology: MDM, IL, JRR. Project administration: MDM, ACJ, EMR, MO, ACC, IL, JRR. Investigation: MDM, ACJ, ACM, ALP, EMR, ACC, MC, MO, ALV, FJAG, PVGP, JG, ARN, FG, CMH, MJV. Formal analysis: MDM, ACJ, ACM, JRR. Resources: MAA, JG, ARN, FG, JLMR, RC, MMT, IL, JRR. Data curation: MDM, ACJ, ACM, ALP, EMR, ACC, MC, MO, MAA, ALV, JG, ARN, FG, CMH, MJV, VMAM, RC. Writing – original draft: MDM, ACJ, ACM, JRR. Writing – review and editing: all authors. Supervision: IL, MMT, JRR. Funding acquisition: IL, MMT, JRR.

## Data availability

Due to privacy concerns, the datasets used in this study are not publicly available. However, researchers can request access to specific individual-level data for academic use only, within 36 months following the publication date, after de-identification. Proposals should be directed to ruizj@ugr.es and idoia.labayen@unavarra.es. Upon proposal acceptance, requestors will be granted access to the data after signing a data access agreement. Raw 16S rRNA gene amplicon sequencing data are available from NCBI Sequence Read Archive (SRA) repository under BioProject PRJNA1287213.

## Financial support

This study (Project ref. PID2022.141506OB.I00) is funded by the MCIU/AEI/10.13039/501100011033 and by ERDF, EU a way of making Europe to JRR; The Junta de Andalucía, Consejería de Transformación económica, Industria, Conocimiento y Universidades (A-CTS-516-UGR20) to JRR; the University of Granada Plan Propio de Investigación-Excellence actions: Unit of Excellence on Exercise Nutrition and Health (UCEENS) to JRR; Spanish Ministry of Universities (FPU18/03357 to MDM, FPU21/01161 to ACJ, and FPU22/01631 to JJMO) – University of Granada; the Government of Navarra, Departamento de Desarrollo Economico y Empresarial (0011-1365-2021-00070), Plan de Promoción de Grupos de Investigación de la Universidad Pública de Navarra , the Spanish Ministry of Science, Innovation, and Universities (MCIU), Instituto de Salud Carlos III (PI24/01360), and by European Regional Development Fund (ERDF), EU a way of making Europe to IL. ALP received a Sara Borrell Contract from the Spanish Ministry of Health (CD22/00109) and a Researcher Contract from Foundation of the Spanish Association Against Cancer (FAECC) (INVES2610029LOPE). ACC is supported by the MCIU, European Union NextGenerationEU/PRTR (FJC2020-043385-I), and by the Junta de Andalucia, Consejeria de Salud y Consumo (RHJ-0098-2024). VMAM is supported by a Margarita Salas grant RCMS-22-KIAO1C-24-GSMTDX, which is funded by the MCIN/AEI/10.13039/501100011033 and the European Union. ACM is supported by JDC2022-048679-I, funded by MCIN/AEI/10.13039/501100011033 and by the European Union “NextGenerationEU”/PRTR. MO is supported by the Spanish Ministry of Economy and Competitiveness (BES-2017-080770). In addition, funding was provided from the EXERNET Research Network on Exercise and Health (DEP2005-00046/ACTI; 09/UPB/19; 45/UPB/20; 27/UPB/21 to IL and JRR).

## Conflict of interest

The authors declare no conflicts of interest.

Please refer to the accompanying ICMJE disclosure forms for further details.
