# Supplementary material for: Time-restricted eating, liver health, and fecal microbiota in adults with overweight or obesity: A randomized controlled trial
Source: JHEP Rep. 2026 Jul 7;8(11):101956. doi: 10.1016/j.jhepr.2026.101956 (PMC13661219; doi:10.1016/j.jhepr.2026.101956)
Supplement: Supplementary file 1 [file mmc1.pdf]

Time-restricted eating, liver health, and fecal microbiota in adults with overweight or obesity: A randomized controlled trial

Manuel Dote-Montero, Antonio Clavero-Jimeno, Adrián Cortés-Martín, Amaya Lopez-Pascual, Elisa Merchan-Ramirez, Alba Camacho-Cardenosa, Mara Concepción, Maddi Osés, Alejandro López-Vázquez, Francisco J. Amaro-Gahete, Juan J. Martin-Olmedo, Lucas Jurado-Fasoli, Alejandro De-la-O, Patricia V. García Pérez, Julio Gálvez, Alba Rodriguez-Nogales, Federico Garcia, Carmen Mbongo Habimana, Marcos Jiménez Vázquez, Víctor Manuel Alfaro-Magallanes, Matías A Avila, Jose L. Martín-Rodríguez, Rafael Cabeza, Manuel Muñoz-Torres, Idoia Labayen, Jonatan R. Ruiz

Table of contents

Supplementary Methods 2
Supplementary Results 6
Supplementary Figures 8
Supplementary Tables 17
Supplementary References 29

Supplementary Methods

Study Assessments and Outcomes

Elastography

Liver stiffness and viscosity were assessed using shear wave elastography and shear wave dispersion, respectively, while liver steatosis was assessed using the attenuation imaging coefficient (ATI). All ultrasound-based assessments were conducted using the Aplio i800 system (Canon Medical Systems Corporation, Tochigi, Japan) under fasting conditions (minimum 4–5 hours).

Liver enzymes

Liver enzyme levels, including alanine aminotransferase (ALT), γ–glutamyltransferase (GGT), and alkaline phosphatase (ALP), were measured from serum samples collected after a 10–12-hour overnight fast using an AU5800 automated analyzer (Beckman Coulter Inc., CA, USA).

Anthropometric

Body weight and height were measured using a calibrated electronic column scale and stadiometer (Seca model 799, Hamburg, Germany).[1] BMI was calculated as weight in kilograms divided by height in meters squared. Waist and hip circumferences were measured following the International Society for the Advancement of Kinanthropometry (ISAK) protocols.[1]

Dietary intake and Mediterranean dietary pattern adherence

Dietary intake was assessed using three non-consecutive 24-hour dietary recalls (two on working days and one on a nonworking day) administered through face-to-face or online interviews conducted by qualified and trained research nutritionists. Energy intake, macronutrient intake, and alcohol intake were calculated from the 24-hour dietary recalls using the EasyDiet-Programa de Gestión de la Consulta® de la Academia Española de Nutrición y Dietética (Biocentury, S.L.U. 2016), computer software supported by the Spanish Association

of Dietitians and Nutritionists. Mediterranean dietary pattern adherence was assessed using the validated 14-item Mediterranean Diet Adherence Screener (MEDAS), with total scores ranging from 0 to 14, where higher scores indicate greater adherence to the Mediterranean dietary pattern.[2]

Insulin resistance

Venous blood samples were collected and stored at −80°C for the analysis of fasting glucose and insulin using automated analyzers as previously described.[3] Then, the homeostasis model assessment of insulin resistance (HOMA-IR) was calculated.[4] Prior to sample collection, participants were instructed to fast for approximately 10 hours, abstain from alcohol and diuretics for 24 hours, avoid stimulants such as caffeine for 12 hours, and refrain from moderate physical activity for 24 hours or vigorous physical activity for 48 hours.

Fecal microbiota

Sequencing was performed on a NextSeq1000-Illumina platform (Illumina Inc., San Diego, CA, USA) with a 2 x 300 bp paired-end read length at the Microbiology Sequencing Service of the *Hospital Universitario Clínico San Cecilio* (Granada, Spain). Bioinformatic analysis of the sequencing data was carried out using the Quantitative Insights Into Microbial Ecology (QIIME2) (v.2024.10) workflow.[5] Briefly, demultiplexed sequences were processed to remove primers sequences using the q2-cutadapt plugin. Denoising to eliminate low-quality bases, dereplication, merging of denoised paired-end reads, chimera removal, and generation of the Amplicon Sequence Variants (ASVs) table were carried out using the DADA2 algorithm.[6] After denoising, an average of 303,426.47 (± 183,940.94, standard deviation) sequences per sample were retained. ASVs were filtered to remove those that appeared in less than 10% of the samples. Taxonomic classification was assigned using a pre-trained classifier based on the SILVA 138.1 SSU reference database,[7] considering the genus-level taxonomy as the highest classification category. Alpha-diversity indices (Shannon entropy, Simpson index, observed

features, and Pielou evenness) and beta-diversity (Bray-Curtis dissimilarity) were calculated using the q2-diversity plugin, with a sampling depth of 38,500 reads per sample. Microbial functions were predicted by Phylogenetic Investigation of Communities by Reconstruction of Unobserved States (PICRUSt2) (v.2.4.2).[8] Absolute counts for taxa and microbial functions were transformed into relative abundance to normalize the data.

Statistical analysis

Regarding fecal microbiota analyses, to examine differences in baseline fecal microbiota between groups, a multivariate analysis of variance (MANOVA) was performed after principal component analysis (PCA) of baseline relative abundances of taxa. The Kruskal-Wallis test, followed by Dunn's *post-hoc* test, was used to compare baseline alpha diversity indices. Differences in beta diversity, based on the Bray-Curtis dissimilarity, were tested using permutational multivariate analysis of variance (PERMANOVA) with 999 permutations. *P* values for both alpha and beta diversity were adjusted using the Bonferroni correction for multiple comparisons. Beta diversity was visualized using principal coordinates analysis (PCoA) plots in all analyses. Within-group differences (i.e., differences between preintervention [PRE] and postintervention [POST] timepoints for each group) were evaluated using the paired t-test or the Wilcoxon signed-rank test for alpha diversity metrics, depending on whether the data were normally distributed or not, and PERMANOVA was used for beta diversity. Linear discriminant analysis effect size (LEfSe)[9] was performed to identify characteristic biomarkers of bacterial taxa and metabolic pathways associated with the intervention within each group by comparing time points (e.g., UC PRE versus UC POST, early TRE PRE versus early TRE POST, etc.). The linear discriminant analysis (LDA) score was set at 2.0. Changes produced in beta diversity during the intervention were tested using PERMANOVA, evaluating the interaction between group and assessment time. Microbiome Multivariable Associations with Lineal Models (MaAsLin3)[10] were conducted to evaluate

changes in taxonomic composition, both in terms of abundance and prevalence, considering the interaction between group and assessment time, adjusting by sex, and including participant ID as a random effect. The UC group was used as the reference group. Multiple testing correction was applied using the Benjamini-Hochberg False Discovery Rate (FDR).[11] Furthermore, we conducted sensitivity and *post-hoc* analyses by repeating the linear mixed-effects models for all outcomes, stratified by sex (i.e., separately for men and women).

Then, we examined the association between changes in hepatic fat fraction and changes in adipokines, hepatokines, adipo-hepatokines, insulin resistance, and fecal microbiota diversity over time using repeated measures correlation analysis, a method that assesses within-individual associations across multiple time points.[12] Pearson's chi-square test was conducted to examine the association between the intervention group and the achievement of a clinically meaningful weight loss (≥5%). We then conducted exploratory analyses comparing changes in hepatic fat fraction and liver-related biomarkers between participants who achieved clinically meaningful weight loss (≥5%; so-called responders) and those who did not (<5%; so-called non-responders). Between-group differences over time were evaluated using repeated-measures linear mixed-effects models that included a group × time interaction term, a random intercept for participant ID, and sex as a covariate. *P* values were corrected for multiple comparisons across outcomes using the Benjamini-Hochberg FDR method.

Linear mixed-effects models fitted using the lme4 package (v.1.1-36), and graphical representations were generated using the ggplot2 package (v.3.5.1). PERMANOVA was performed using the vegan package (v.2.6.10) and the adonis2 function using default settings (999 permutations). Repeated measures correlation analyses were assessed using the rmcorr package in R (v.0.7.0).

Supplementary Results

Dietary intake and adherence to Mediterranean Dietary Pattern

As reported before,[13] the decrease in energy intake was significantly greater in the early TRE group (mean difference: -304 kcal/day; 95% CI, -565 to -44; $P = 0.01$) compared with the UC group (**Table 2**). No other significant between-group differences were observed for changes in energy intake, macronutrient intake, or alcohol intake (all $P \geq 0.09$**; Tables 2 and 3**).

Mediterranean dietary pattern adherence, assessed using the 14-item MEDAS, showed mean (SD) baseline scores of 6.7 (1.9) in the UC group, 6.8 (2.1) in the early TRE group, 7.2 (1.8) in the late TRE group, and 6.8 (1.5) in the self-selected TRE group. After the 12-week intervention, no significant between-group differences were observed in changes in MEDAS score (all $P \geq 0.54$).

Correlations between changes in hepatic fat fraction, fecal microbiota, and insulin resistance

Changes in hepatic fat fraction were not associated with changes in fecal microbiota diversity metrics or composition (all $P \geq 0.22$). In addition, when TRE participants were grouped according to the baseline presence of MASLD, no differences were found in changes in fecal microbiota diversity or composition throughout the intervention (all $P \geq 0.65$).

As reported before,[13] no statistically significant differences were detected in changes in insulin resistance, as assessed by HOMA-IR, across all groups after the intervention (all $P \geq 0.86$). However, we observed that reductions in hepatic fat fraction after the intervention were significantly associated with decreases in HOMA-IR across all sample (r = 0.416; $P < 0.001$; **Table S2**), and in TRE groups alone (r = 0.360, $P < 0.001$; **Table S2**).

Eating window times and adherence

As reported before,[13] the eating window was significantly shorter in the early TRE group (median: 7.7 h; interquartile range, 0.5; $P < 0.001$), the late TRE group (median: 7.4 h;

interquartile range, 0.8; $P < 0.001$), and the self-selected TRE group (median: 7.6 h; interquartile range, 0.4; $P < 0.001$) compared to the UC group (median: 13.4 h; interquartile range, 1.2). Median eating window times were 08:30 to 22:00 for the UC group, 09:45 to 17:30 for the early TRE group, 14:20 to 21:30 for the late TRE group, and 12:20 to 20:00 for the self-selected TRE group. The percentage of adherent days (defined as an eating window ≤9 h) was comparable across the early TRE group (mean: 85.3%; 95% CI, 80.6 to 90.0), late TRE group (mean: 88.1%; 95% CI, 84.9 to 91.3) and self-selected TRE group (mean: 85.4%; 95% CI, 81.3 to 89.5) (all $P \geq 0.99$).

Sensitivity analyses: effects of the intervention by sex

Of note is that we repeated all the analyses separately in men and women, and the results consistently showed similar effects across the TRE groups compared with UC and among the TRE groups themselves (data not shown).

Supplementary Figures

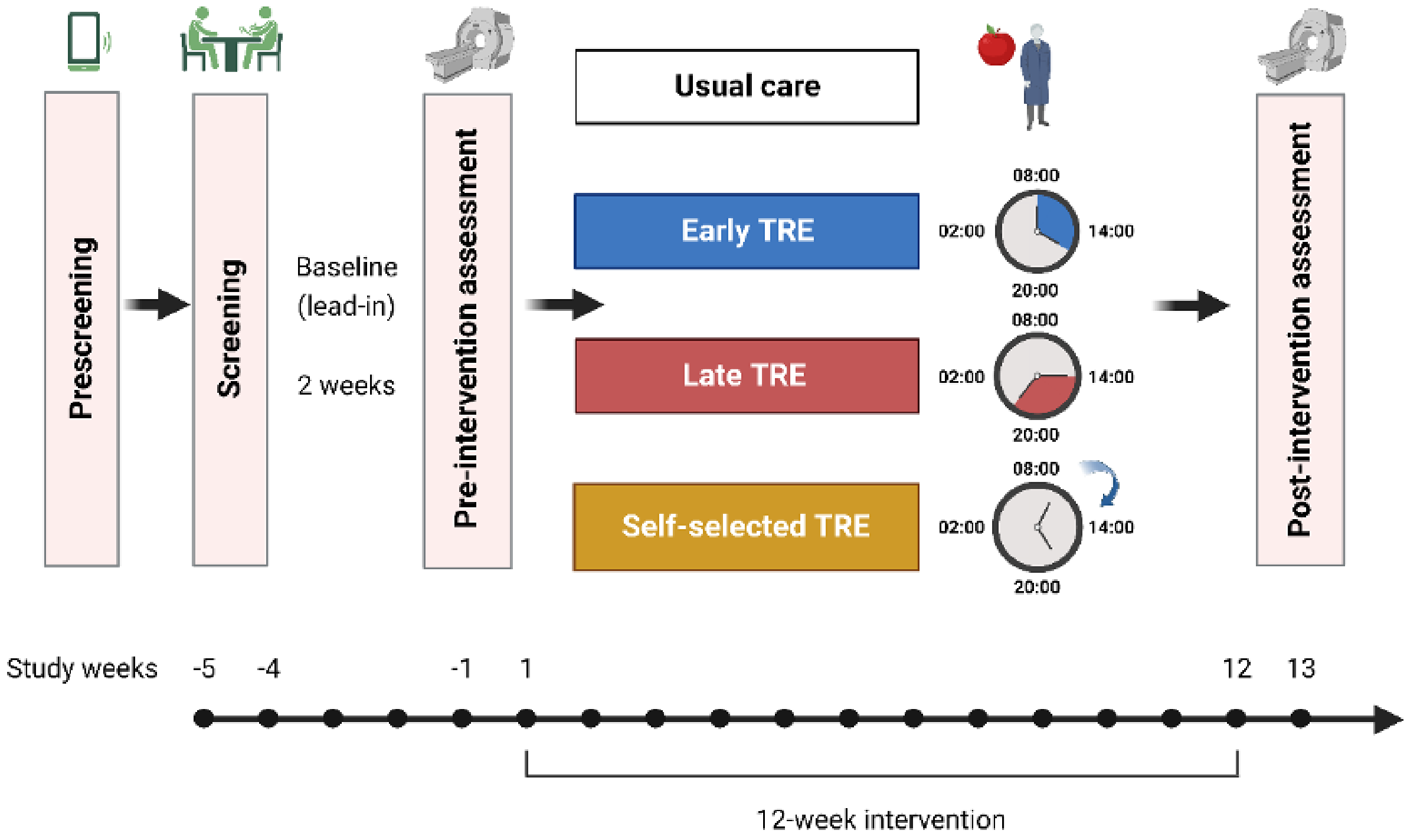

Fig. S1. Study design. Prescreening of potential participants was performed by phone calls, and potential participant were scheduled for a screening visit. The randomized controlled trial included a 2-week baseline (lead-in period) and 12-week dietary intervention. TRE, time-restricted eating.

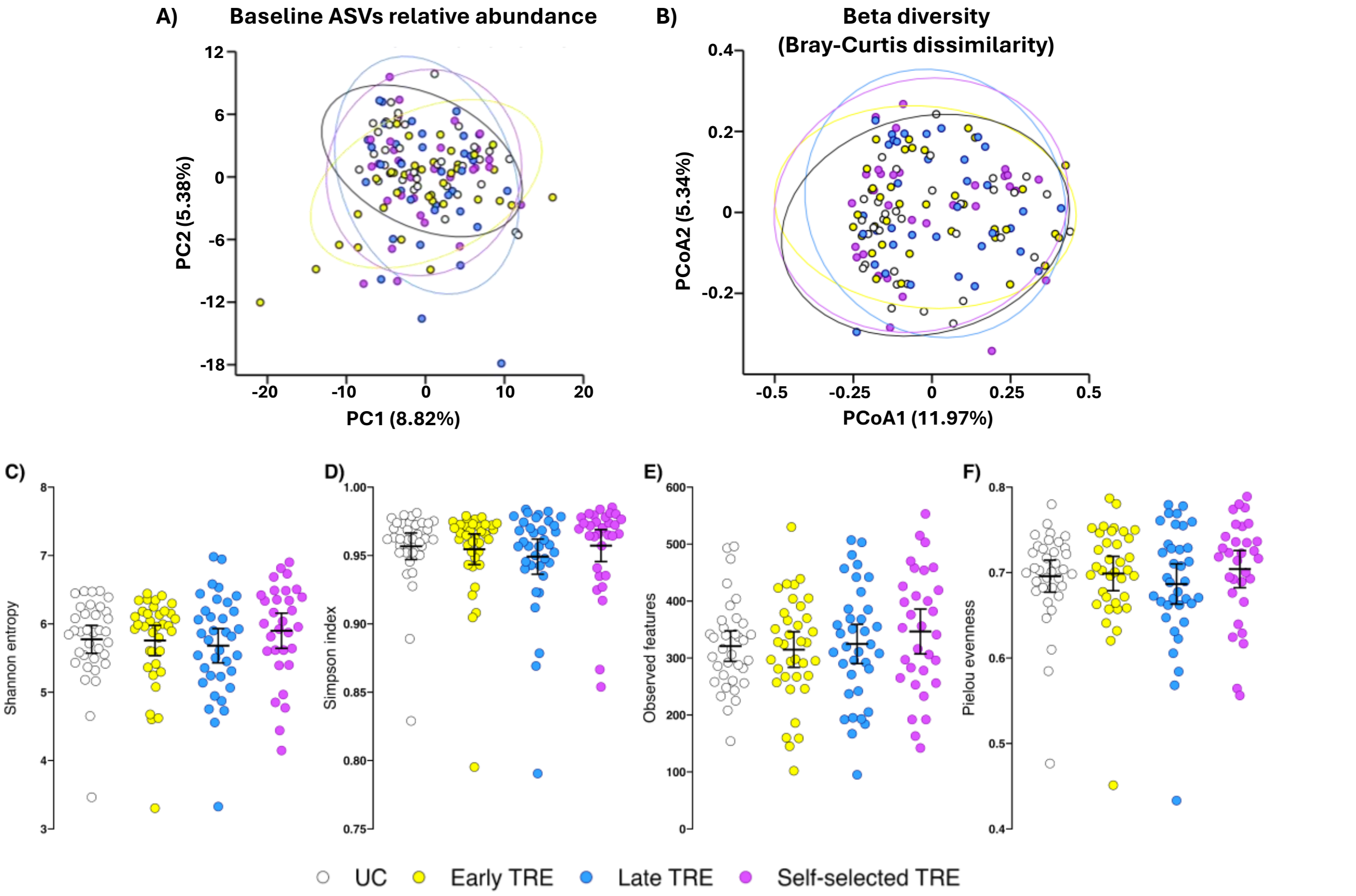

Fig. S2. Baseline gut microbiota composition and diversity in the the usual care (UC), early time–restricted eating (TRE), late TRE, and self–selected TRE groups. **(A)** Principal component analysis (PCA) plot of the baseline relative abundance of the amplicon sequence variants (ASVs) detected through 16S rRNA sequencing (*P* = 0.29). **(B)** Principal coordinate analysis (PCoA) plot based on Bray-Curtis distance of baseline gut microbiota composition (*P* = 0.30). **(C-F)** Comparison of baseline alpha-diversity metrics between groups: **(C)** Shannon entropy (*P* = 0.42), **(D)** Simpson index (*P* = 0.45), **(E)** observed features (*P* = 0.63), and **(F)** Pielou evenness (*P* = 0.65). Statistical analyses were performed using MANOVA and PERMANOVA for PCA and PCoA analyses, respectively, and the Kruskal-Wallis test for the comparisons between groups for alpha-diversity indices. Significant differences were set at *P* < 0.05.

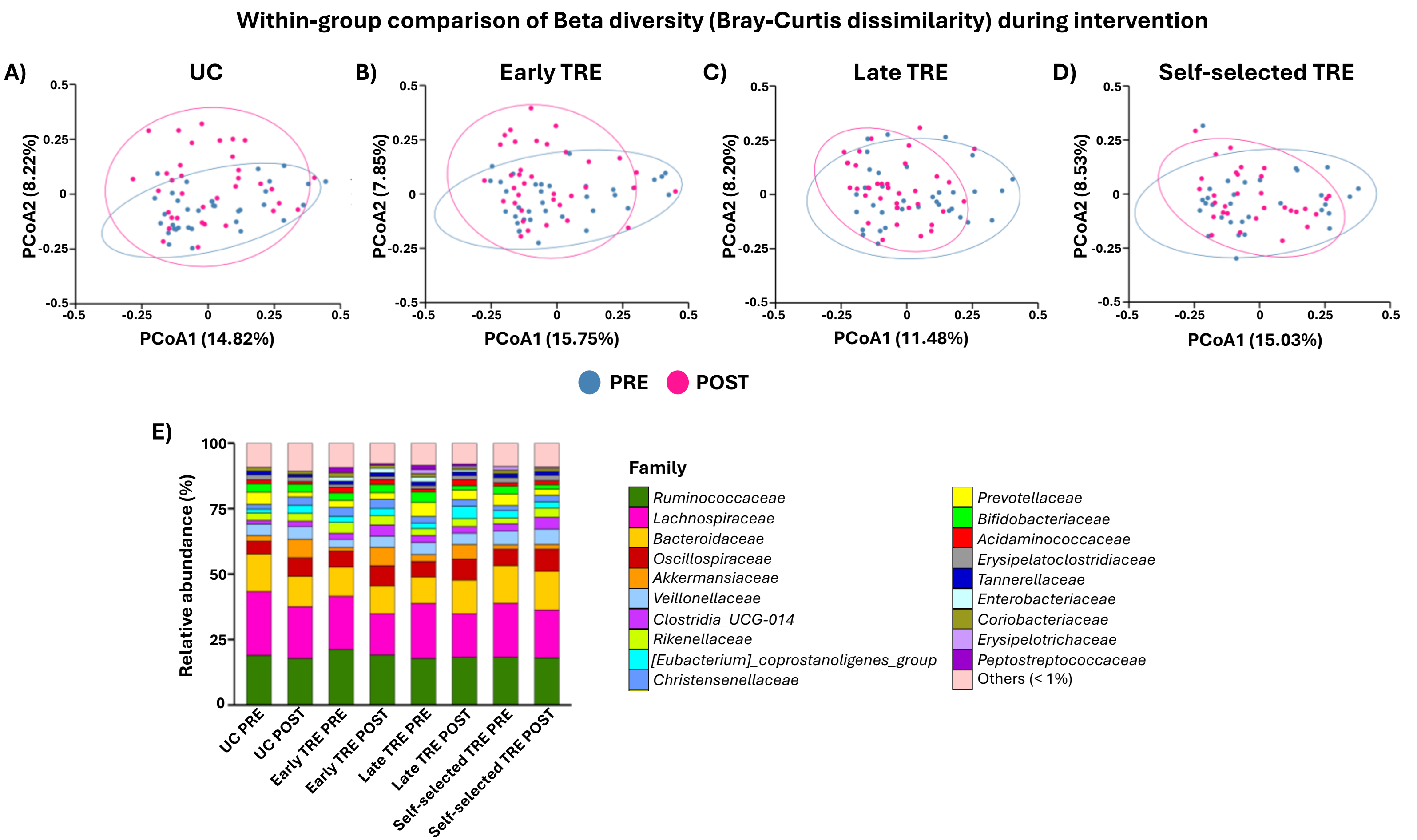

Fig. S3. **(A-D)** PCoA plots representing within-group comparisons of beta-diversity (Bray-Curtis dissimilarity) at the two-time points of the intervention, PRE (blue) and POST (pink), for the usual care (UC; A; P = 0.26), early time–restricted eating (TRE; B; P = 0.10), late TRE (C; P = 0.19), and self–selected TRE (D; P = 0.76) groups. Statistical analyses for PCoA were performed using PERMANOVA. **(E)** Microbial taxonomic composition at the family level in fecal samples for the PRE and POST time points for each intervention group. The bars show the mean proportion of relative abundance. The "Others" category in the graph represents the aggrupation of bacterial groups with less than 1% relative abundance. Changes in bacterial composition were evaluated through MaAsLin3 algorithm considering the assigned group, assessment time, and their interactions terms with sex as a covariate. No statistically significant differences were detected after applying Benjamini-Hochberg False Discovery Rate (FDR). Significant differences were set at $P < 0.05$.

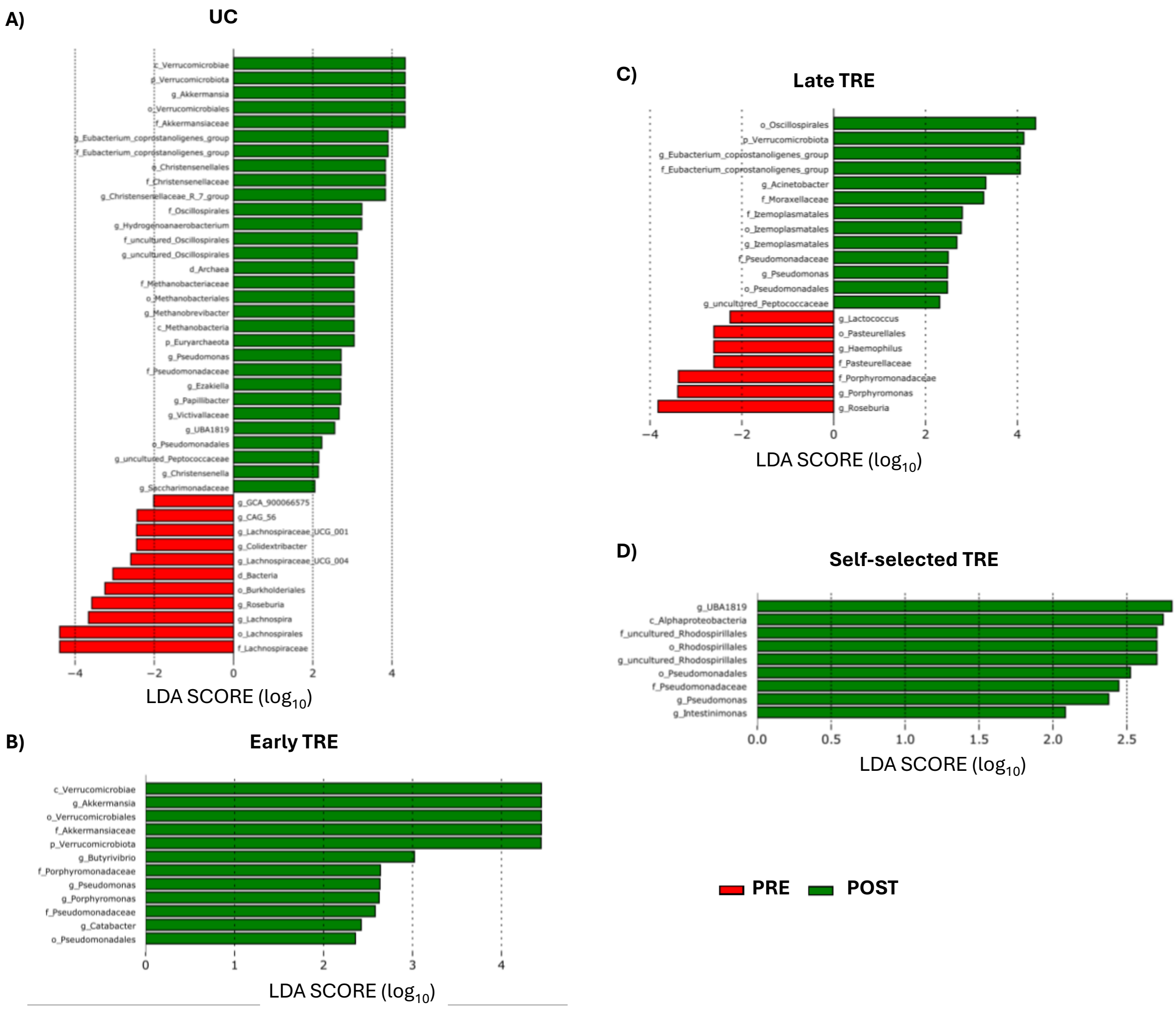

Fig. S4. Linear discriminant analysis effect size (LEfSe) for taxa biomarker discovery between preintervention (PRE) and postintervention (POST) within the usual care (UC; A), early time–restricted eating (TRE; B), late TRE (C), and self–selected TRE (D) groups. Histograms of the Linear Discriminant Analysis (LDA) scores show features that are differentially abundant between PRE (red) and POST (green) in each intervention group. The LDA score threshold was set to 2.0, and significant differences were set at $P < 0.05$.

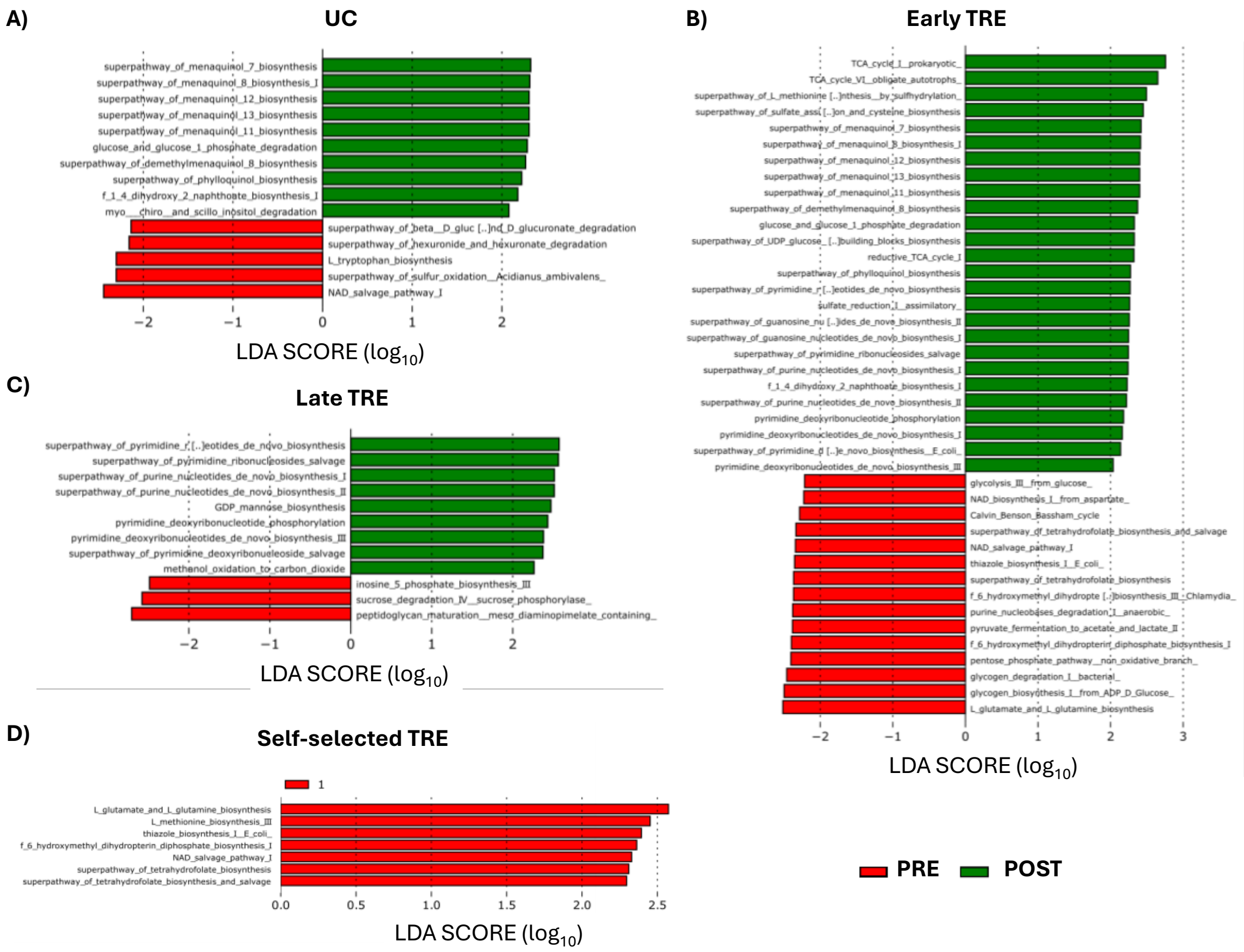

Fig. S5. Linear discriminant analysis effect size (LEfSe) for metabolic pathway biomarker discovery between PRE and POST time points within the usual care (UC; A), early time–restricted eating (TRE; B), late TRE (C), and self–selected TRE (D) groups. Histograms of the Linear Discriminant Analysis (LDA) scores show differentially abundant bacterial pathways between preintervention (PRE; red) and postintervention (POST; green) in each intervention group. The LDA score threshold was set to 2.0, and significant differences were set at *P* < 0.05.

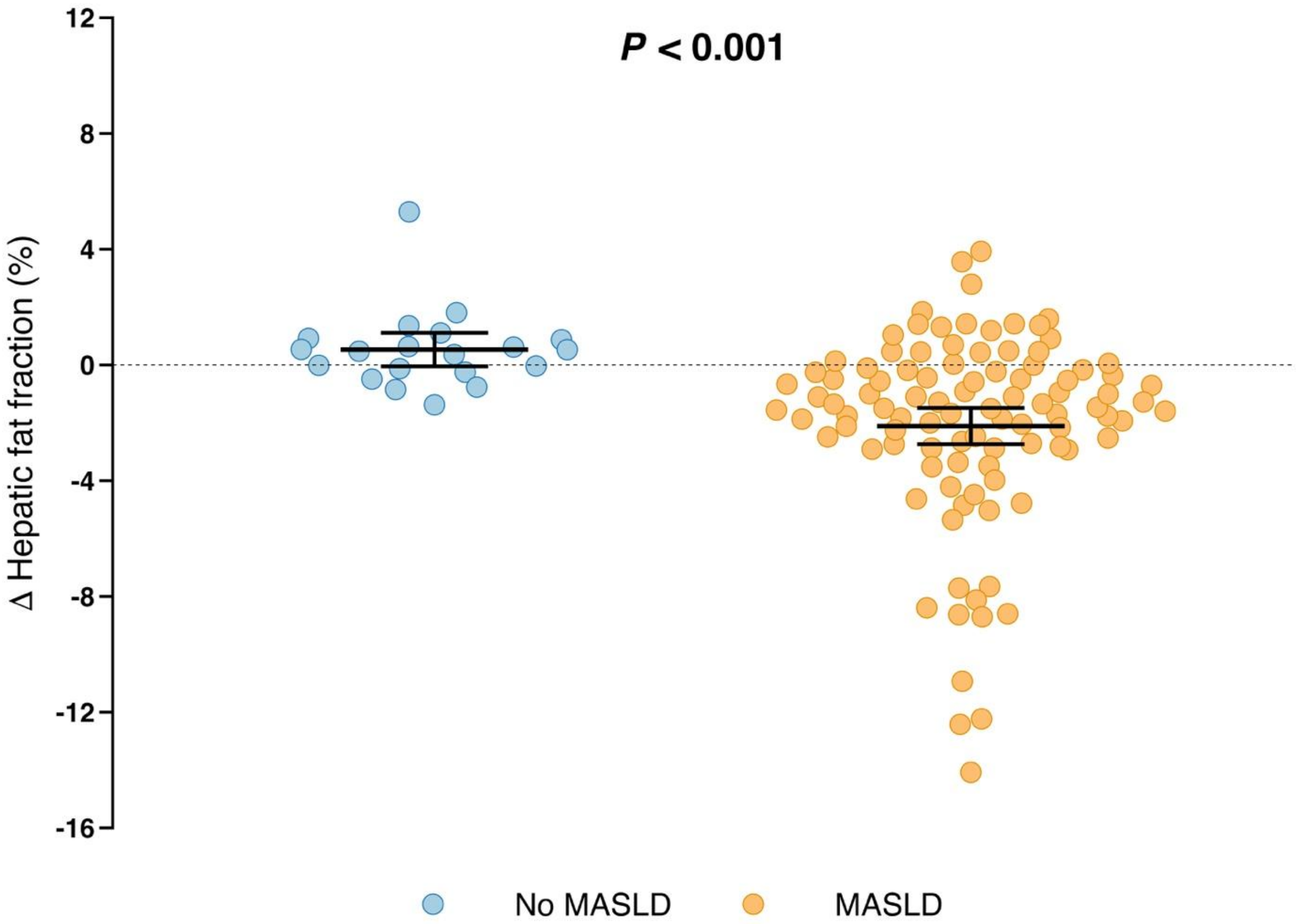

Fig. S6. Changes in hepatic fat fraction in participants from the time-restricted eating groups, stratified by the presence of metabolic dysfunction-associated steatotic liver disease (MASLD) at baseline (defined as ≥5% hepatic fat fraction) (n = 111) compared to those without the condition (defined as <5% hepatic fat fraction) (n = 24). Data are raw means with 95% confidence interval. Between-group differences (No MASLD vs. MASLD) were evaluated using linear mixed-effects models with repeated measures adjusted for sex. Statistically significant differences are displayed in bold ($P < 0.05$). Hepatic fat fraction was assessed by magnetic resonance imaging. Changes were calculated as postintervention minus preintervention values.

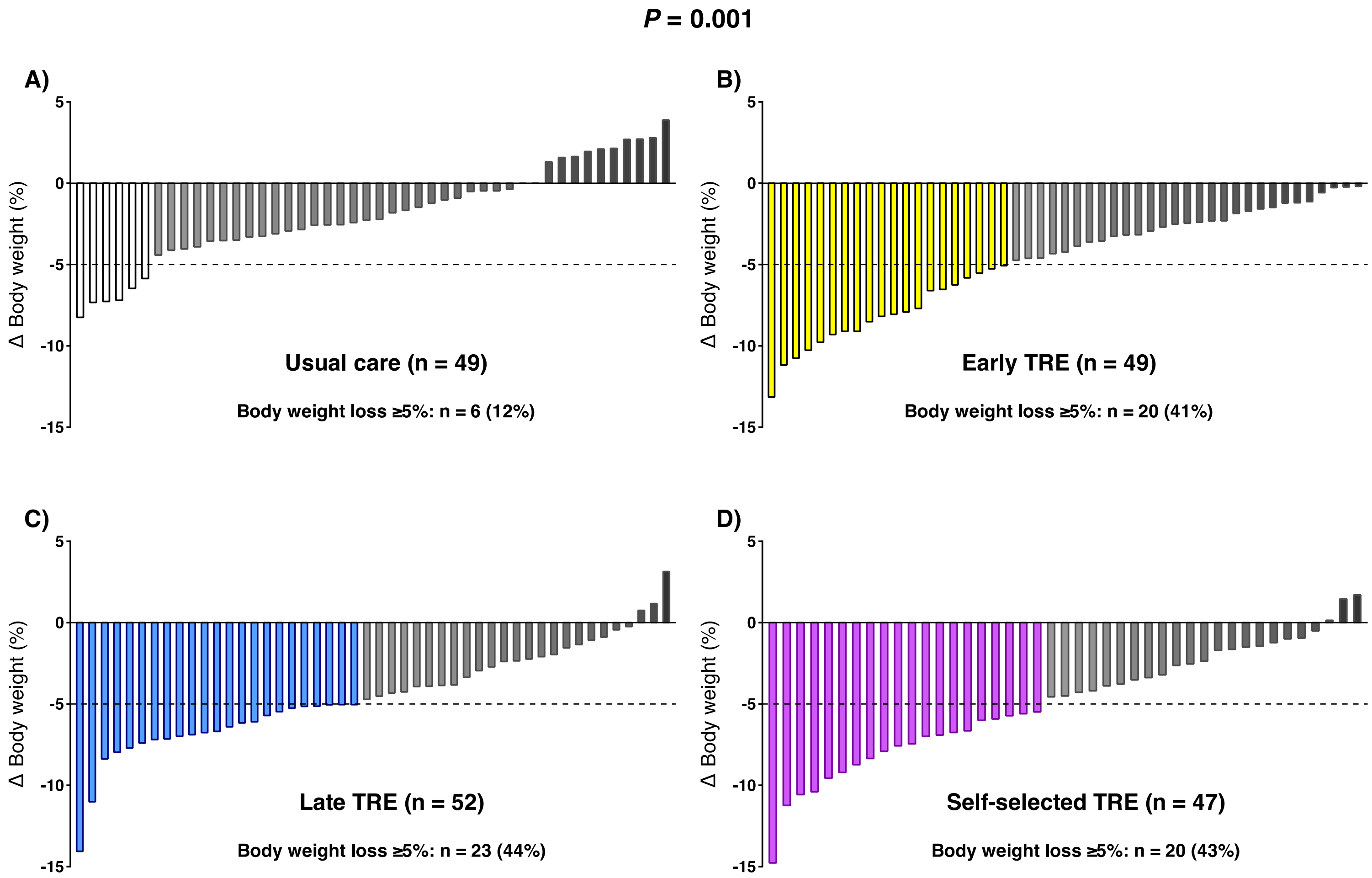

Fig. S7. Changes in body weight percentage among the usual care (A), early time–restricted eating (TRE; B), late TRE (C), and self–selected TRE (D) groups after the 12-week intervention. The columns represent the raw data of individual study participants. Bars in color indicate participants who achieved clinically meaningful weight loss (≥5%), while gray bars indicate those who did not (<5%). The dotted line represents the 5% weight loss threshold. *P* value from Pearson's chi-square. Body weight percentages indicate changes relative to preintervention values.

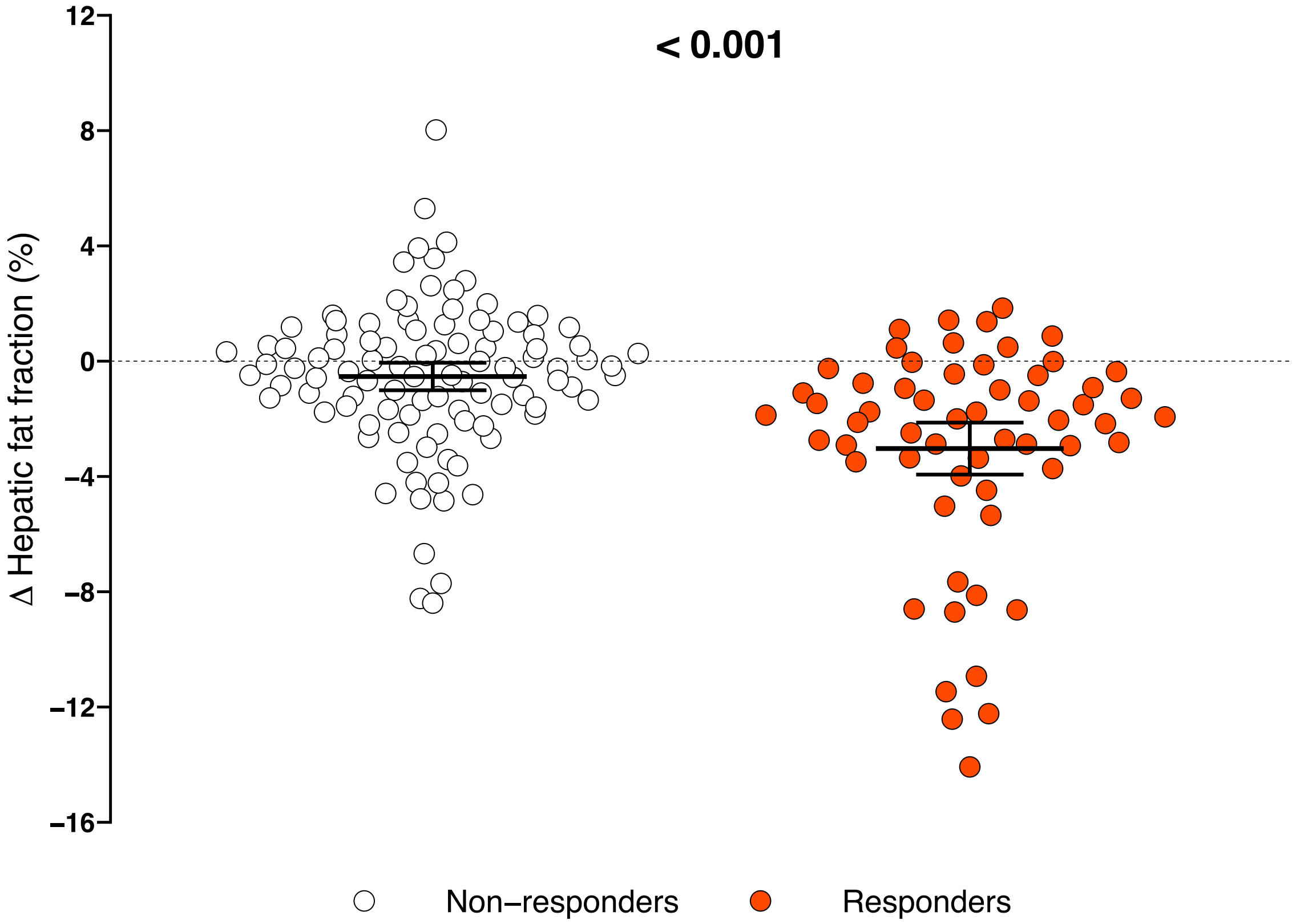

Fig. S8. Changes in hepatic fat fraction in participants who did not achieve clinically significant body weight loss (<5%, namely non-responders) compared to those who achieved a clinically significant body weight loss (≥5%, namely responders). Data are raw means with 95% confidence interval. Between-group differences (non-responders vs. responders) were evaluated using linear mixed-effects models with repeated measures adjusted for sex. *P* value was corrected using the Benjamini-Hochberg False Discovery Rate (FDR) method to control for multiple comparisons across outcomes. Statistically significant differences are displayed in bold (FDR-adjusted $P < 0.05$). Hepatic fat fraction was assessed by magnetic resonance imaging. Changes were calculated as postintervention minus preintervention values.

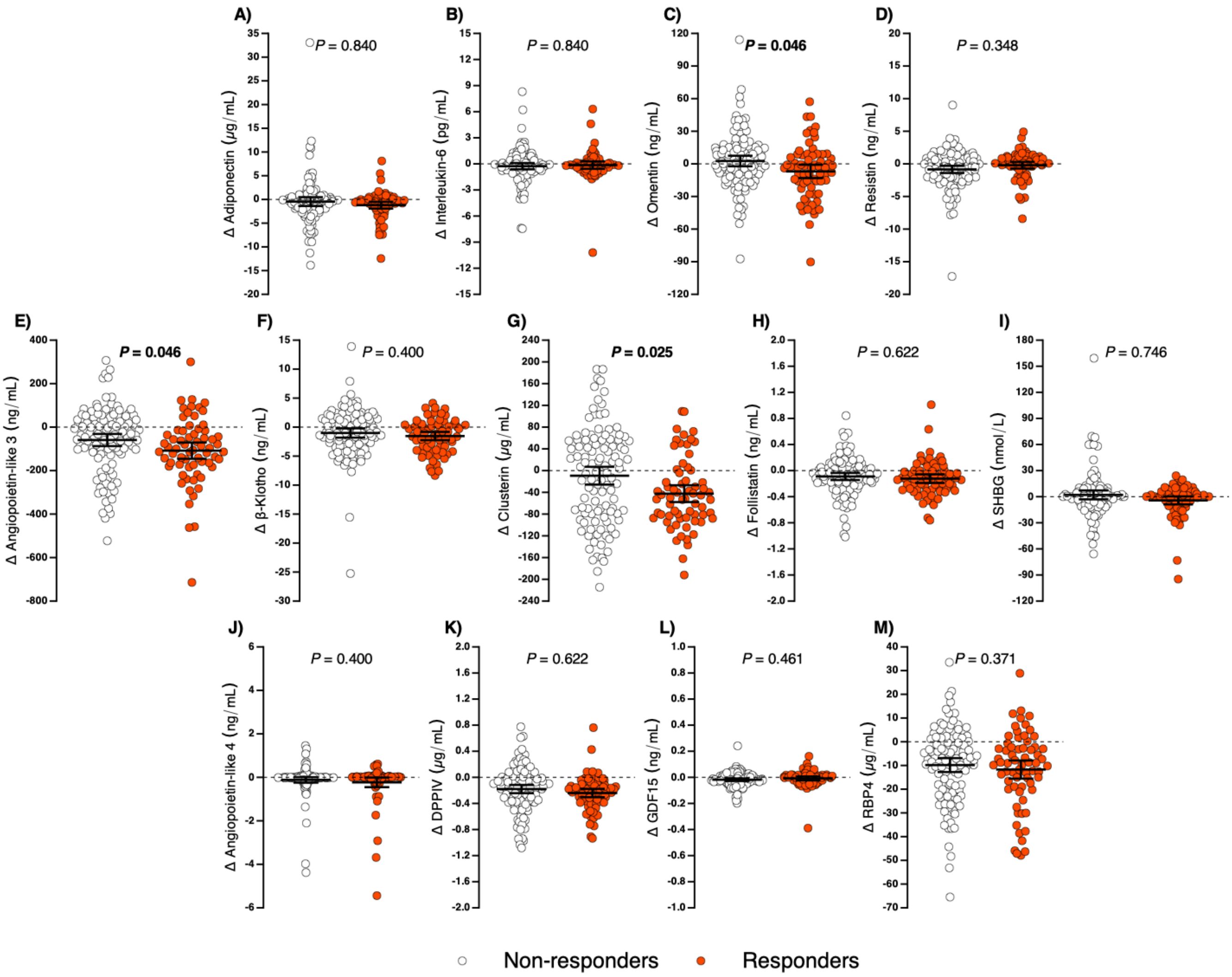

Fig. S9. Changes in adiponectin (A), interleukin-6 (B), omentin (C), resistin (D), angiopoietin-like 3 (E), β-Klotho (F), clusterin (G), follistatin (H), sex hormone-binding globulin (SHBG; I), angiopoietin-like 4 (J), dipeptidyl peptidase IV (DPPIV; K), growth differentiation factor 15 (GDF15; L), and retinol-binding protein 4 (RBP4; M) in participants who did not achieve clinically significant body weight loss (<5%, namely non-responders) compared to those who achieved a clinically significant body weight loss (≥5%, namely responders). Data are raw means with 95% confidence interval. Between-group differences (non-responders vs. responders) were evaluated using linear mixed-effects models with repeated measures adjusted for sex. *P* values were corrected using the Benjamini-Hochberg False Discovery Rate (FDR) method to control for multiple comparisons across outcomes. Statistically significant differences are displayed in bold (FDR-adjusted $P < 0.05$). Adipokines, hepatokines, and adipo-hepatokines were log10-transformed for statistical analyses; however, results are presented in the original units for improved interpretability. Changes were calculated as postintervention minus preintervention values.

Supplementary Tables

Table S1. Liver parameters, adipokines, hepatokines, adipo-hepatokines, and dietary intake endpoints at baseline and after the 12–week intervention in each intervention group.

**Endpoint**	**UC**		**Early TRE**		**Late TRE**		**Self-selected TRE**	
	n	Mean (95% CI)	n	Mean (95% CI)	n	Mean (95% CI)	n	Mean (95% CI)
Liver parameters								
Hepatic fat fraction (%)								
Preintervention	47	10.9 (9.4, 12.3)	44	8.8 (7.4, 10.3)	48	10.7 (9.3, 12.1)	43	8.5 (7.0, 9.9)
Postintervention	42	10.1 (8.6, 11.5)	41	7.7 (6.2, 9.1)	46	8.4 (7.0, 9.8)	39	7.0 (5.4, 8.5)
Change	48	-0.8 (-1.8, 0.2)	46	-1.2 (-2.2, -0.2) *	51	-2.3 (-3.3, -1.3) *	45	-1.5 (-2.6, -0.5) *
Liver stiffness (kPa)								
Preintervention	38	7.1 (6.3, 7.9)	34	7.1 (6.3, 7.9)	36	7.5 (6.7, 8.3)	31	6.9 (6.0, 7.7)
Postintervention	35	6.7 (5.9, 7.5)	37	7.1 (6.3, 7.9)	40	7.6 (6.9, 8.4)	35	6.6 (5.7, 7.4)
Change	43	-0.4 (-1.2, 0.4)	42	0.0 (-0.8, 0.8)	46	0.2 (-0.6, 1.0)	43	-0.3 (-1.2, 0.6)
ATI (dB/cm/MHz)								
Preintervention	38	0.71 (0.68, 0.75)	35	0.72 (0.69, 0.76)	36	0.74 (0.71, 0.77)	32	0.70 (0.66, 0.73)
Postintervention	35	0.70 (0.67, 0.73)	37	0.66 (0.62, 0.69)	40	0.69 (0.66, 0.72)	35	0.65 (0.62, 0.68)
Change	43	-0.01 (-0.04, 0.02)	43	-0.07 (-0.10, -0.04) *	46	-0.05 (-0.08, -0.02) *	43	-0.04 (-0.08, -0.01) *
Viscosity (m/s/kHz)								
Preintervention	25	11.1 (9.9, 12.2)	23	11.8 (10.6, 13.0)	26	11.1 (10.0, 12.2)	21	10.8 (9.6, 12.1)
Postintervention	24	11.0 (9.8, 12.2)	23	11.1 (9.9, 12.3)	26	11.5 (10.4, 12.7)	20	11.1 (9.9, 12.4)
Change	25	-0.1 (-1.5, 1.4)	23	-0.7 (-2.2, 0.8)	27	0.4 (-1.0, 1.8)	22	0.3 (-1.2, 1.9)
ALT (U/L)								
Preintervention	49	30 (25, 34)	49	28 (24, 33)	52	28 (24, 33)	47	27 (23, 32)
Postintervention	44	28 (24, 33)	47	25 (21, 30)	48	24 (19, 28)	43	22 (18, 27)
Change	49	-2 (-6, 2)	49	-3 (-7, 1)	52	-5 (-9, -1) *	47	-5 (-9, -1) *
GGT (U/L)								
Preintervention	49	48 (35, 61)	49	28 (15, 41)	52	35 (22, 47)	47	29 (16, 43)
Postintervention	44	44 (31, 57)	47	25 (12, 38)	48	31 (18, 43)	43	25 (12, 38)
Change	49	-5 (-11, 2)	49	-3 (-10, 3)	52	-4 (-10, 2)	47	-4 (-11, 2)
ALP (U/L)								
Preintervention	49	66 (61, 70)	49	65 (61, 70)	52	68 (64, 73)	47	66 (61, 71)
Postintervention	44	66 (61, 71)	47	68 (63, 73)	48	67 (63, 72)	43	66 (61, 71)
Change	49	0 (-2, 3)	49	3 (0, 6) *	52	-1 (-4, 2)	47	0 (-3, 3)
Adipokines								
Adiponectin (µg/mL)								
Preintervention	47	7.6 (6.1, 9.0)	47	7.8 (6.4, 9.3)	51	5.7 (4.3, 7.1)	46	7.0 (5.5, 8.5)
Postintervention	43	6.8 (5.4, 8.3)	47	6.6 (5.2, 8.1)	48	5.3 (3.9, 6.7)	43	6.5 (5.0, 8.0)
Change	49	-0.7 (-2.0, 0.6) *	49	-1.2 (-2.5, 0.1)	52	-0.4 (-1.7, 0.9)	47	-0.5 (-1.8, 0.8)
Interleukin–6 (pg/mL)								
Preintervention	47	2.2 (1.8, 2.7)	45	2.5 (2.1, 3.0)	49	2.7 (2.2, 3.1)	45	2.2 (1.7, 2.7)
Postintervention	44	2.3 (1.8, 2.7)	47	2.3 (1.8, 2.7)	48	2.2 (1.7, 2.6)	43	2.0 (1.5, 2.4)
Change	48	0.0 (-0.5, 0.6)	48	-0.3 (-0.8, 0.3)	50	-0.5 (-1.0, 0.0) *	47	-0.2 (-0.8, 0.3)
Omentin (ng/mL)								
Preintervention	47	79.5 (63.4, 95.6)	45	56.4 (40.2, 72.5)	51	50.3 (34.7, 65.8)	47	55.2 (38.8, 71.5)
Postintervention	43	75.6 (59.4, 91.9)	47	53.1 (37.0, 69.1)	48	55.0 (39.4, 70.7)	43	53.8 (37.3, 70.3)
Change	49	-3.9 (-12.0, 4.2)	49	-3.3 (-11.2, 4.6)	52	4.8 (-2.8, 12.4)	47	-1.4 (-9.3, 6.5)
Resistin (ng/mL)								
Preintervention	47	4.6 (4.0, 5.3)	47	4.4 (3.7, 5.1)	51	4.4 (3.7, 5.0)	47	4.8 (4.1, 5.5)
Postintervention	43	3.8 (3.1, 4.5)	47	4.4 (3.7, 5.0)	48	3.5 (2.9, 4.2)	43	4.0 (3.3, 4.7)
Change	49	-0.8 (-1.6, 0.0)	49	0.0 (-0.9, 0.8)	52	-0.8 (-1.6, -0.1)	47	-0.8 (-1.6, 0.1)
Hepatokines								
Angiopoietin–like 3 (ng/mL)								
Preintervention	47	455.1 (416.9, 493.3)	47	445.6 (407.4, 483.9)	50	419.7 (382.6, 456.8)	46	393.3 (354.6, 432.0)
Postintervention	43	351.0 (311.2, 390.8)	47	369.3 (331.0, 407.5)	48	358.5 (320.8, 396.3)	43	331.8 (291.9, 371.6)

Change	49	-104.1 (-150.6, -57.6) *	49	-76.4 (-121.5, -31.2) *	52	-61.2 (-105.6, -16.8) *	47	-61.5 (-108.1, -15.0) *
β–Klotho (ng/mL)								
Preintervention	47	12.6 (11.1, 14.1)	45	10.9 (9.4, 12.4)	51	11.0 (9.6, 12.5)	47	11.5 (10.0, 13.0)
Postintervention	43	10.6 (9.1, 12.1)	47	9.6 (8.1, 11.1)	47	9.8 (8.4, 11.3)	43	11.1 (9.5, 12.6)
Change	49	-2.1 (-3.2, -0.9) *	49	-1.3 (-2.5, -0.1) *	52	-1.2 (-2.3, -0.1) *	47	-0.5 (-1.6, 0.7)
Clusterin (µg/mL)								
Preintervention	47	228.7 (213.1, 244.3)	47	237.9 (222.3, 253.6)	50	231.8 (216.6, 246.9)	46	228.8 (213.0, 244.6)
Postintervention	43	208.5 (192.2, 224.8)	47	216.3 (200.7, 232.0)	48	210.1 (194.6, 225.5)	43	204.3 (188.0, 220.6)
Change	49	-20.2 (-42.8, 2.5) *	49	-21.6 (-43.8, 0.5) *	52	-21.7 (-43.4, -0.0) *	47	-24.5 (-47.3, -1.7) *
Follistatin (ng/mL)								
Preintervention	47	1.02 (0.91, 1.14)	46	1.03 (0.92, 1.14)	49	1.01 (0.90, 1.12)	47	0.94 (0.82, 1.05)
Postintervention	43	0.92 (0.81, 1.04)	47	0.91 (0.80, 1.03)	48	0.92 (0.80, 1.03)	43	0.85 (0.73, 0.96)
Change	49	-0.10 (-0.19, -0.01)	48	-0.12 (-0.20, -0.03) *	50	-0.10 (-0.18, -0.01) *	47	-0.09 (-0.17, -0.00) *
Sex Hormone-Binding Globulin (nmol/L)								
Preintervention	44	25.9 (20.5, 31.3)	47	22.3 (17.1, 27.5)	49	24.1 (18.9, 29.2)	45	20.5 (15.1, 25.8)
Postintervention	42	21.2 (15.7, 26.7)	43	24.6 (19.1, 30.1)	47	19.0 (13.8, 24.2)	42	22.5 (17.0, 28.0)
Change	48	-4.8 (-12.4, 2.9)	48	2.3 (-5.2, 9.8)	51	-5.0 (-12.3, 2.2)	47	2.0 (-5.6, 9.6)
Adipo-hepatokines								
Angiopoietin–like 4 (ng/mL)								
Preintervention	47	1.28 (0.89, 1.67)	46	0.56 (0.16, 0.95)	49	0.81 (0.43, 1.20)	47	0.84 (0.45, 1.24)
Postintervention	43	0.96 (0.56, 1.35)	47	0.46 (0.07, 0.86)	48	0.76 (0.38, 1.15)	42	0.68 (0.28, 1.08)
Change	49	-0.32 (-0.56, -0.08)	48	-0.09 (-0.32, 0.14) *	50	-0.05 (-0.28, 0.17)	47	-0.17 (-0.40, 0.07)
Dipeptidyl Peptidase IV (µg/mL)								
Preintervention	47	1.07 (0.98, 1.15)	47	1.04 (0.95, 1.13)	51	1.01 (0.93, 1.10)	46	0.96 (0.88, 1.05)
Postintervention	43	0.85 (0.76, 0.94)	47	0.79 (0.70, 0.87)	48	0.86 (0.77, 0.94)	43	0.80 (0.71, 0.89)
Change	49	-0.22 (-0.31, -0.12) *	49	-0.25 (-0.34, -0.16) *	52	-0.16 (-0.25, -0.07) *	47	-0.16 (-0.26, -0.07) *
Growth Differentiation Factor 15 (ng/mL)								
Preintervention	47	0.28 (0.26, 0.31)	47	0.29 (0.27, 0.32)	50	0.29 (0.27, 0.32)	47	0.25 (0.22, 0.28)
Postintervention	43	0.27 (0.24, 0.30)	47	0.27 (0.24, 0.29)	48	0.28 (0.25, 0.30)	43	0.25 (0.22, 0.27)
Change	49	-0.01 (-0.03, 0.00) *	49	-0.03 (-0.04, -0.01) *	51	-0.02 (-0.03, -0.00) *	47	0.00 (-0.02, 0.02)
Retinol-Binding Protein 4 (µg/mL)								
Preintervention	47	35.6 (31.8, 39.5)	47	36.9 (33.0, 40.8)	50	35.8 (32.1, 39.6)	46	34.8 (30.9, 38.7)
Postintervention	43	25.8 (21.8, 29.9)	47	26.1 (22.2, 30.0)	48	24.8 (21.0, 28.7)	43	25.0 (21.0, 29.1)
Change	49	-9.8 (-14.6, -5.0) *	49	-10.8 (-15.5, -6.2) *	52	-11.0 (-15.6, -6.4) *	47	-9.8 (-14.6, -5.0) *
Dietary intake								
Energy intake (kcal/day)								
Preintervention	49	2034 (1910, 2158)	49	2024 (1899, 2148)	52	1874 (1754, 1995)	47	1995 (1868, 2122)
Postintervention	46	1807 (1679, 1935)	48	1492 (1367, 1618)	48	1503 (1378, 1628)	43	1525 (1393, 1657)
Change	49	-227 (-368, -86) *	49	-531 (-671, -392) *	52	-372 (-510, -234) *	47	-470 (-616, -324) *
Carbohydrate intake (% EI)								
Preintervention	49	36 (33, 38)	49	35 (33, 37)	52	35 (33, 37)	47	37 (35, 40)
Postintervention	46	35 (33, 38)	48	35 (33, 38)	48	35 (33, 37)	43	37 (34, 39)
Change	49	0 (-3, 2)	49	1 (-2, 3)	52	0 (-2, 2)	47	-1 (-3, 2)
Fat intake (% EI)								
Preintervention	49	43 (41, 45)	49	44 (42, 46)	52	45 (43, 47)	47	43 (41, 45)
Postintervention	46	43 (41, 45)	48	43 (41, 45)	48	43 (41, 45)	43	42 (40, 44)
Change	49	0 (-3, 2)	49	-1 (-4, 1)	52	-2 (-4, 1)	47	-1 (-4, 1)
Protein intake (% EI)								
Preintervention	49	18 (17, 19)	49	17 (17, 18)	52	18 (17, 19)	47	17 (16, 18)
Postintervention	46	19 (18, 20)	48	18 (17, 19)	48	19 (18, 20)	43	19 (18, 20)
Change	49	1 (0, 2)	49	1 (0, 2)	52	1 (0, 2) *	47	2 (0, 3) *
Alcohol intake (% EI)								
Preintervention	49	3 (2, 4)	49	3 (2, 4)	52	2 (1, 3)	47	2 (1, 3)
Postintervention	46	3 (2, 4)	48	3 (2, 4)	48	3 (2, 4)	43	3 (2, 4)
Change	49	0 (-1, 1)	49	0 (-1, 1)	52	1 (0, 2)	47	0 (-1, 1)

Data are presented as estimated marginal means with 95% confidence intervals (CIs) from linear mixed models. These are model-derived estimates, not raw means, which explains any differences with baseline values in Table 1. Asterisks (*) represent statistically significant within-group changes as determined by linear mixed model contrasts ($P < 0.05$). No adjustments

were made for multiple comparisons within groups. Hepatic fat fraction was assessed by magnetic resonance imaging. Liver stiffness, attenuation imaging coefficient (ATI), and viscosity were assessed using elastography. Adipokines, hepatokines, and adipo-hepatokines were log10-transformed for statistical analyses; however, results are presented in original units to improve interpretability. As a result, some CIs may appear to include zero or not in the original scale, although all analyses were performed on the log10-transformed data. Dietary intake was estimated from three non-consecutive 24–hour dietary recalls, two on working days and one on a non-working day. Changes were calculated as postintervention minus preintervention values. The number of participants with valid data at each time point (Preintervention, Postintervention) and in the final model (Change) is reported by group for each variable. "Change" reflects the total sample included in the linear mixed models, which account for missing data and may include participants with only Preintervention, only Postintervention, or both measurements. ALT, alanine aminotransferase; ALP, alkaline phosphatase; GGT, γ–glutamyltransferase; TRE, time–restricted eating; UC, usual care.

Table S2. Associations between changes in hepatic fat fraction and changes in adipokines, hepatokines, adipo-hepatokines, and insulin resistance after the 12-week intervention in all sample (i.e., including usual care, early time-restricted eating [TRE], late TRE, and self-selected TRE groups), and in TRE groups alone.

	**All sample**		**TRE groups alone**	
	r_{rm} (95% CI)	*P* value	r_{rm} (95% CI)	*P* value
Adipokines				
Adiponectin (µg/mL)	0.033 (-0.127, 0.191)	0.684	-0.006 (-0.189, 0.177)	0.947
Interleukin–6 (pg/mL)	-0.028 (-0.187, 0.133)	0.733	-0.029 (-0.213, 0.158)	0.763
Omentin (ng/mL)	0.104 (-0.056, 0.260)	0.202	0.078 (-0.107, 0.259)	0.407
Resistin (ng/mL)	0.024 (-0.136, 0.182)	0.773	0.016 (-0.167, 0.197)	0.868
Hepatokines				
Angiopoietin–like 3 (ng/mL)	0.263 (0.107, 0.405)	**0.001**	0.242 (0.061, 0.408)	**0.009**
β–Klotho (ng/mL)	0.274 (0.119, 0.416)	**0.001**	0.288 (0.109, 0.449)	**0.002**
Clusterin (µg/mL)	0.264 (0.109, 0.407)	**0.001**	0.222 (0.039, 0.390)	**0.018**
Follistatin (ng/mL)	0.264 (0.109, 0.405)	**0.001**	0.302 (0.127, 0.459)	**0.001**
Sex Hormone-Binding Globulin (nmol/L)	0.087 (-0.078, 0.248)	0.300	0.040 (-0.151, 0.227)	0.684
Adipo-hepatokines				
Angiopoietin–like 4 (ng/mL)	-0.072 (-0.228, 0.087)	0.375	-0.065 (-0.244, 0.119)	0.488
Dipeptidyl Peptidase IV (µg/mL)	0.307 (0.156, 0.445)	**< 0.001**	0.263 (0.084, 0.426)	**0.005**
Growth Differentiation Factor 15 (ng/mL)	0.033 (-0.126, 0.191)	0.685	0.030 (-0.153, 0.211)	0.747
Retinol-Binding Protein 4 (µg/mL)	0.402 (0.258, 0.527)	**< 0.001**	0.387 (0.219, 0.533)	**< 0.001**
Insulin resistance				
HOMA-IR	0.416 (0.276, 0.540)	**< 0.001**	0.360 (0.188, 0.511)	**< 0.001**

Repeated measures correlation (r_{rm}) was used to assess longitudinal associations between within-individual changes in hepatic fat fraction and changes in adipokines, hepatokines, adipohepatokines, and insulin resistance. Positive r_{rm} values indicate that reductions in hepatic fat fraction were associated with decreases in the respective biomarker or insulin resistance, whereas negative values suggest that reductions in hepatic fat fraction are associated with increases in the respective biomarker or insulin resistance. Adipokines, hepatokines, and adipo-hepatokines were log10-transformed for the present statistical analyses. Confidence intervals (CIs) were calculated using an analytic approach with a 95% confidence level. Significant associations are indicated in bold ($P < 0.05$). HOMA-IR, homeostasis model assessment of insulin resistance.

Table S3. Alpha diversity indices of gut microbiota before and after the intervention in the usual care (UC), early time–restricted eating (TRE), late TRE, and self–selected TRE groups.

Endpoint	UC (n = 35)			Early TRE (n = 36)			Late TRE (n = 36)			Self-selected TRE (n = 31)		
	Before	After	*P*	Before	After	*P*	Before	After	*P*	Before	After	*P*
**Shannon entropy**	5.88 (5.52 – 6.16)	5.66 (5.28 – 6.10)	0.20	5.97 (5.55 – 6.16)	5.78 (5.44 – 6.08)	0.43	5.79 (5.24 – 6.18)	5.95 (5.51 – 6.25)	0.90	6.00 (5.61 – 6.41)	6.00 (5.57 – 6.20)	0.72
**Simpson index**	0.96 (0.95 – 0.97)	0.95 (0.93 – 0.97)	**0.02**	0.97 (0.95 – 0.97)	0.96 (0.94 – 0.97)	0.25	0.96 (0.94 – 0.97)	0.96 (0.94 – 0.97)	0.66	0.97 (0.95 – 0.98)	0.97 (0.95 – 0.98)	0.99
**Observed features**	321 (78)	340 (103)	0.27	315 (92)	346 (74)	0.06	325 (102)	355 (91)	0.10	347 (108)	342 (92)	0.79
**Pielou evenness**	0.70 (0.68 – 0.73)	0.68 (0.65 – 0.70)	**0.02**	0.70 (0.67 – 0.74)	0.69 (0.65 – 0.72)	0.10	0.69 (0.66 – 0.73)	0.69 (0.65 – 0.73)	0.26	0.72 (0.68 – 0.74)	0.71 (0.68 – 0.74)	0.87

Data are presented as mean (standard deviation) when normally distributed, or medians (first quartile – third quartile) when not. Within-group comparisons were performed using the t paired t-test or the Wilcoxon signed-rank test, depending on whether the data were normally distributed or not, respectively. Significant differences are indicated in bold ($P < 0.05$).

Table S4. Baseline characteristics of participants classified as non-responders (i.e., body weight loss <5%) and responders (i.e., body weight loss ≥5%).

	**Non-responders (n = 117)**	**Responders (n = 69)**
Age (years)	46.9 (6.6)	46.9 (5.8)
Women, No. (%)	57 (48.7)	31 (44.9)
Weight (kg)	94.3 (16.2)	97.6 (11.6)
Height (cm)	169.4 (9.4)	170.8 (8.4)
Body mass index (kg/m^2)	32.7 (3.9)	33.4 (2.7)
Waist circumference (cm)	100.9 (10.4)	103.7 (9.1)
Hip circumference (cm)	113.9 (8.6)	115.1 (7.3)
Waist to hip ratio	0.9 (0.1)	0.9 (0.1)
Liver parameters		
Hepatic fat fraction (%)	7.5 (5.6 – 11.5)	8.6 (5.7 – 11.6)
MASLD prevalence, No. (%)[a]	93 (82.3)	53 (86.9)
Stage of hepatic steatosis[a]		
S0, No. (%)	20 (17.7)	8 (13.1)
S1, No. (%)	79 (69.9)	42 (68.9)
S2, No. (%)	12 (10.6)	8 (13.1)
S3, No. (%)	2 (1.8)	3 (4.9)
Liver stiffness (kPa)	6.7 (5.8 – 7.6)	6.3 (5.2 – 7.5)
ATI (dB/cm/MHz)	0.70 (0.64 – 0.76)	0.70 (0.64 – 0.76)
Viscosity (m/s/kHz)	10.4 (9.2 – 12.3)	11.2 (9.3 – 12.6)
ALT (U/L)	23 (17 – 32)	29 (21 – 39)
GGT (U/L)	25 (18 – 36)	26 (19 – 42)
ALP (U/L)	61 (53 – 75)	66 (57 – 78)
Adipokines		
Adiponectin (µg/mL)	6.3 (4.2 – 9.3)	5.6 (3.4 – 7.1)
Interleukin–6 (pg/mL)	2.1 (1.4 – 2.9)	1.9 (1.5 – 2.6)
Omentin (ng/mL)	49.4 (35.7 – 65.3)	53.2 (35.0 – 69.7)
Resistin (ng/mL)	4.3 (2.8 – 6.0)	3.8 (2.6 – 4.8)
Hepatokines		
Angiopoietin–like 3 (ng/mL)	386.2 (312.9 – 501.3)	412.0 (319.5 – 489.8)
β–Klotho (ng/mL)	10.9 (9.7 – 11.9)	10.6 (9.9 – 11.9)
Clusterin (µg/mL)	222.0 (195.5 – 260.7)	231.6 (203.3 – 261.3)
Follistatin (ng/mL)	0.98 (0.78 – 1.17)	0.90 (0.73 – 1.13)
Sex Hormone–Binding Globulin (nmol/L)	19.6 (14.9 – 27.3)	17.5 (10.6 – 28.5)
Adipo–hepatokines		
Angiopoietin–like 4 (ng/mL)	0.28 (0.17 – 1.18)	0.21 (0.15 – 0.70)
Dipeptidyl Peptidase IV (µg/mL)	0.96 (0.79 – 1.24)	0.96 (0.85 – 1.13)
Growth Differentiation Factor 15 (ng/mL)	0.27 (0.23 – 0.34)	0.25 (0.22 – 0.30)
Retinol–Binding Protein 4 (µg/mL)	34.1 (24.3 – 46.9)	29.7 (21.9 – 43.4)
Dietary intake		
Energy intake (kcal/day)	1941 (1620 – 2242)	1960 (1718 – 2271)
Carbohydrate intake (% EI)	36 (31 – 40)	36 (32 – 39)
Fat intake (% EI)	17 (15 – 20)	17 (15 – 20)
Protein intake (% EI)	44 (40 – 47)	44 (40 – 48)
Alcohol intake (% EI)	2 (0 – 4)	1 (0 – 3)
Cardiovascular risk factors		
Systolic BP (mm Hg)	123 (14)	124 (14)
Diastolic BP (mm Hg)	81 (10)	80 (11)
Fasting glucose (mg/dL)	92 (88 – 98)	94 (90 – 100)
Fasting insulin (mU/L)	10.2 (7.6 – 13.5)	10.9 (8.8 – 15.1)
HOMA–IR	2.46 (1.64 – 3.18)	2.53 (1.99 – 3.80)
HbA1c (%)	5.3 (5.1 – 5.5)	5.4 (5.2 – 5.6)
Total cholesterol (mg/dL)	210 (184 – 228)	207 (186 – 231)
HDL–C (mg/dL)	54 (45 – 61)	51 (45 – 57)
LDL–C (mg/dL)	127 (111 – 147)	136 (116 – 156)

Triglycerides (mg/dL)	123 (82 – 159)	114 (90 – 149)

Data are presented as mean (standard deviation) when normally distributed, median (first quartile – third quartile) when not normally distributed, or as frequency (associated percentage). Hepatic fat fraction was assessed by magnetic resonance imaging (MRI). Metabolic dysfunction-associated steatotic liver disease (MASLD) prevalence was defined as the number and percentage of participants with hepatic fat fraction ≥5%. Stages of hepatic steatosis were defined as follows: S0: no steatosis [hepatic fat fraction <5%]; S1: mild steatosis [hepatic fat fraction ≥5% and <15%]; S2: moderate steatosis [hepatic fat fraction ≥15% and <25%]; S3: severe steatosis [hepatic fat fraction ≥25%].[14] Liver stiffness, attenuation imaging coefficient (ATI), and viscosity were assessed using elastography. Dietary intake was estimated from three non-consecutive 24–hour dietary recalls, two on working days and one on a non-working day. No statistically significant differences were observed at baseline for any outcome across groups (analysis of variance with Benjamini–Hochberg adjustment; all $P \geq 0.31$). ALT, alanine aminotransferase; ALP, alkaline phosphatase; APOA1, apolipoprotein A; APOB, apolipoprotein B; BP, blood pressure; EI, energy intake; GGT, γ–glutamyltransferase; HbA1c, glycated hemoglobin; HDL–C, high–density lipoprotein cholesterol; HOMA–IR, homeostasis model assessment of insulin resistance; LDL–C, low–density lipoprotein cholesterol; TRE, time–restricted eating; UC, usual care. [a]Calculated based on the maximum number of participants with available hepatic fat fraction data.

Table S5. Liver parameters, adipokines, hepatokines, adipo-hepatokines, and dietary intake endpoints at baseline and after the 12–week intervention in non-responders (i.e., body weight loss <5%) and responders (i.e., body weight loss ≥5%).

**Endpoint**	**Non-responders (n = 117)**		**Responders (n = 69)**		**Responders vs. non-responders**	
	n	Mean (95% CI)	n	Mean (95% CI)	Mean difference (95% CI)	*P* value
Liver parameters						
Hepatic fat fraction (%)						
Preintervention	113	9.4 (8.5, 10.3)	61	10.2 (9.0, 11.5)		
Postintervention	103	8.9 (8.0, 9.9)	65	7.2 (5.9, 8.4)		
Change	115	-0.5 (-1.1, 0.1)	67	-3.1 (-3.8, -2.3) *	-2.6 (-3.6, -1.6)	**<0.001**
Liver stiffness (kPa)						
Preintervention	84	7.1 (6.5, 7.6)	47	7.2 (6.5, 7.9)		
Postintervention	98	6.9 (6.4, 7.4)	49	7.2 (6.5, 7.9)		
Change	105	-0.2 (-0.7, 0.3)	61	0.0 (-0.7, 0.8)	0.2 (-0.7, 1.1)	0.798
ATI (dB/cm/MHz)						
Preintervention	84	0.72 (0.70, 0.74)	50	0.72 (0.69, 0.75)		
Postintervention	98	0.68 (0.66, 0.70)	49	0.67 (0.64, 0.70)		
Change	105	-0.04 (-0.06, -0.02) *	63	-0.05 (-0.08, -0.02) *	-0.01 (-0.05, 0.02)	0.724
Viscosity (m/s/kHz)						
Preintervention	64	11.1 (10.4, 11.9)	27	11.3 (10.2, 12.4)		
Postintervention	66	11.2 (10.5, 11.9)	27	11.1 (10.0, 12.2)		
Change	66	0.1 (-0.8, 1.0)	27	-0.2 (-1.6, 1.2)	-0.3 (-2.0, 1.3)	0.831
ALT (U/L)						
Preintervention	117	27 (25, 30)	69	30 (26, 34)		
Postintervention	115	27 (24, 30)	67	21 (17, 25)		
Change	117	0 (-3, 2)	69	-9 (-12, -6) *	-9 (-13, -5)	**<0.001**
GGT (U/L)						
Preintervention	117	38 (29, 47)	69	31 (19, 42)		
Postintervention	115	35 (27, 44)	67	24 (12, 35)		
Change	117	-3 (-7, 1)	69	-7 (-12, -2) *	-4 (-11, 2)	0.400
ALP (U/L)						
Preintervention	117	65 (62, 68)	69	68 (64, 72)		
Postintervention	115	67 (64, 70)	67	67 (63, 71)		
Change	117	2 (0, 4)	69	-1 (-3, 1)	-3 (-6, 0)	0.206
Adipokines						
Adiponectin (µg/mL)						
Preintervention	111	7.5 (6.5, 8.4)	69	6.4 (5.2, 7.6)		
Postintervention	114	7.0 (6.1, 7.9)	67	5.2 (4.0, 6.4)		
Change	117	-0.5 (-1.3, 0.4)	69	-1.2 (-2.3, -0.2)	-0.8 (-2.1, 0.6)	0.840
Interleukin–6 (pg/mL)						
Preintervention	111	2.5 (2.2, 2.8)	66	2.2 (1.8, 2.6)		
Postintervention	115	2.2 (1.9, 2.5)	67	2.1 (1.7, 2.4)		
Change	116	-0.3 (-0.6, 0.1) *	68	-0.2 (-0.6, 0.3)	0.1 (-0.5, 0.7)	0.840
Omentin (ng/mL)						
Preintervention	111	62.7 (51.9, 73.4)	68	58.2 (44.3, 72.2)		
Postintervention	114	65.4 (54.7, 76.1)	67	51.2 (37.2, 65.2)		
Change	117	2.8 (-2.2, 7.7)	69	-7.0 (-13.4, -0.7) *	-9.8 (-17.8, -1.8)	**0.046**
Resistin (ng/mL)						
Preintervention	112	4.9 (4.5, 5.4)	69	4.0 (3.5, 4.6)		
Postintervention	114	4.0 (3.6, 4.5)	67	3.8 (3.2, 4.4)		
Change	117	-0.9 (-1.4, -0.4) *	69	-0.2 (-0.9, 0.5)	0.7 (-0.2, 1.5)	0.348
Hepatokines						
Angiopoietin–like 3 (ng/mL)						
Preintervention	111	426.4 (401.2, 451.5)	68	438.1 (405.9, 470.4)		
Postintervention	114	367.7 (342.8, 392.5)	67	330.6 (298.1, 363.0)		
Change	117	-58.7 (-87.7, -29.7) *	69	-107.5 (-144.9, -70.2) *	-48.8 (-96.2, -1.5)	**0.046**

β–Klotho (ng/mL)						
Preintervention	111	11.5 (10.6, 12.5)	68	11.2 (10.0, 12.3)		
Postintervention	113	10.5 (9.6, 11.4)	67	9.6 (8.4, 10.8)		
Change	117	-1.0 (-1.8, -0.3) *	69	-1.5 (-2.5, -0.6) *	-0.5 (-1.7, 0.7)	0.400
Clusterin (μg/mL)						
Preintervention	111	231.9 (222.0, 241.9)	68	234.1 (221.4, 246.8)		
Postintervention	114	221.2 (211.3, 231.0)	67	190.8 (178.0, 203.7)		
Change	117	-10.8 (-24.8, 3.2) *	69	-43.3 (-61.4, -25.2) *	-32.5 (-55.4, -9.6)	**0.025**
Follistatin (ng/mL)						
Preintervention	112	1.02 (0.95, 1.10)	68	0.96 (0.86, 1.05)		
Postintervention	114	0.94 (0.86, 1.01)	67	0.83 (0.74, 0.93)		
Change	117	-0.09 (-0.14, -0.03) *	68	-0.12 (-0.19, -0.05) *	-0.04 (-0.12, 0.05)	0.622
Sex Hormone-Binding Globulin (nmol/L)						
Preintervention	107	22.8 (19.5, 26.2)	67	22.1 (17.8, 26.4)		
Postintervention	110	23.8 (20.5, 27.1)	64	18.1 (13.7, 22.5)		
Change	116	1.0 (-3.7, 5.7)	67	-4.0 (-10.1, 2.0)	-5.0 (-12.7, 2.7)	0.746
Adipo-hepatokines						
Angiopoietin–like 4 (ng/mL)						
Preintervention	112	0.89 (0.64, 1.15)	68	0.91 (0.58, 1.25)		
Postintervention	113	0.78 (0.52, 1.04)	67	0.68 (0.35, 1.02)		
Change	117	-0.12 (-0.26, 0.03) *	68	-0.23 (-0.42, -0.04)	-0.11 (-0.35, 0.13)	0.400
Dipeptidyl Peptidase IV (μg/mL)						
Preintervention	111	1.04 (0.99, 1.10)	69	1.00 (0.93, 1.07)		
Postintervention	114	0.87 (0.81, 0.92)	67	0.76 (0.69, 0.83)		
Change	117	-0.18 (-0.24, -0.12) *	69	-0.24 (-0.32, -0.17) *	-0.06 (-0.16, 0.03)	0.622
Growth Differentiation Factor 15 (ng/mL)						
Preintervention	112	0.29 (0.27, 0.31)	69	0.26 (0.24, 0.28)		
Postintervention	114	0.27 (0.26, 0.29)	67	0.25 (0.23, 0.28)		
Change	117	-0.02 (-0.03, -0.01) *	69	-0.01 (-0.02, 0.01)	0.01 (-0.01, 0.03)	0.461
Retinol-Binding Protein 4 (μg/mL)						
Preintervention	111	37.4 (34.9, 39.9)	68	33.8 (30.6, 37.0)		
Postintervention	114	27.4 (24.9, 29.8)	67	22.3 (19.1, 25.5)		
Change	117	-10.0 (-13.0, -7.0) *	69	-11.5 (-15.3, -7.7) *	-1.5 (-6.3, 3.4)	0.371
Dietary intake						
Energy intake (kcal/day)						
Preintervention	117	1966 (1884, 2048)	69	2022 (1916, 2128)		
Postintervention	117	1622 (1541, 1704)	68	1516 (1409, 1623)		
Change	117	-343 (-434, -253) *	69	-506 (-624, -389) *	-163 (-311, -15)	0.131
Carbohydrate intake (% EI)						
Preintervention	117	35 (34, 37)	69	36 (34, 37)		
Postintervention	117	36 (34, 37)	68	35 (33, 37)		
Change	117	0 (-1, 2)	69	0 (-2, 1)	-1 (-3, 2)	0.798
Fat intake (% EI)						
Preintervention	117	44 (42, 45)	69	45 (43, 46)		
Postintervention	117	43 (41, 44)	68	43 (42, 45)		
Change	117	-1 (-3, 1)	69	-1 (-3, 1)	0 (-3, 2)	0.840
Protein intake (% EI)						
Preintervention	117	18 (17, 18)	69	18 (17, 18)		
Postintervention	117	18 (18, 19)	68	19 (18, 20)		
Change	117	1 (0, 2) *	69	2 (1, 3) *	1 (0, 2)	0.400
Alcohol intake (% EI)						
Preintervention	117	3 (2, 4)	69	2 (1, 3)		
Postintervention	117	3 (3, 4)	68	2 (1, 3)		
Change	117	0 (0, 1)	69	0 (-1, 1)	0 (-1, 1)	0.840

Data for preintervention, postintervention, and change are presented as estimated marginal means with 95% confidence intervals (CIs) from linear mixed models. These are model-derived estimates, not raw means, which explains any differences with baseline values in Table S4. Asterisks (*) represent statistically significant within-group changes as determined by linear mixed model contrasts ($P < 0.05$). Data for between-group differences (non-responders vs. responders) are presented as estimated mean differences and 95% CIs and were evaluated using linear mixed-effects models with repeated measures, adjusted for sex. *P* values were corrected using the Benjamini-Hochberg False Discovery Rate (FDR) method to

control for multiple comparisons across outcomes. Differences are reported as responders minus non-responders. Statistically significant differences are displayed in bold (FDR-adjusted $P < 0.05$). Participants classified as non-responders did not achieve clinically significant body weight loss (<5%), while responders achieved ≥5% body weight loss. All participants from usual care (UC), early time-restricted eating (TRE), late TRE, and self-selected TRE groups with available body weight data at both preintervention and postintervention assessments were included in the present analyses. Sample sizes for each category are as follows: UC = 40 non-responders, and 6 responders; early TRE = 29 non-responders, and 20 responders; late TRE = 25 non-responders, and 23 responders; self-selected TRE = 23 non-responders, and 20 responders. Hepatic fat fraction was assessed by magnetic resonance imaging. Liver stiffness, attenuation imaging coefficient (ATI), and viscosity were assessed using elastography. Adipokines, hepatokines, and adipo-hepatokines were log10-transformed for statistical analyses; however, results are presented in original units to improve interpretability. As a result, some confidence intervals may appear to include zero or not in the original scale, although all analyses were performed on the log10-transformed data. Dietary intake was estimated from three non-consecutive 24–hour dietary recalls, two on working days and one on a non-working day. Changes were calculated as postintervention minus preintervention values. The number of participants with valid data at each time point (Preintervention, Postintervention) and in the final model (Change) is reported by group for each variable. "Change" reflects the total sample included in the linear mixed models, which account for missing data and may include participants with only Preintervention, only Postintervention, or both measurements. ALT, alanine aminotransferase; ALP, alkaline phosphatase; EI, energy intake; GGT, γ–glutamyltransferase.

Table S6. CONSORT 2025 checklist of information to include when reporting a randomized trial.

Section/Topic	Item No	CONSORT 2025 checklist item description	Reported on page No
		**Title and abstract**	
Title and structured abstract	1a	Identification as a randomized trial	1 & 7
	1b	Structured summary of the trial design, methods, results, and conclusions	7
Trial registration	2	Name of trial registry, identifying number (with URL) and date of registration	7 & 11
Protocol and statistical analysis plan	3	Where the trial protocol and statistical analysis plan can be accessed	11
Data sharing	4	Where and how the individual de-identified participant data (including data dictionary), statistical code and any other materials can be accessed	25
Funding and conflicts of interest	5a	Sources of funding and other support (eg, supply of drugs), and role of funders in the design, conduct, analysis and reporting of the trial	3-4
	5b	Financial and other conflicts of interest of the manuscript authors	3-4
Background and rationale	6	Scientific background and rationale	9-10
Objectives	7	Specific objectives related to benefits and harms	10
Patient and public involvement	8	Details of patient or public involvement in the design, conduct and reporting of the trial	11-12
Trial design	9	Description of trial design including type of trial (eg, parallel group or crossover), allocation ratio, and framework (for example, superiority, equivalence, non-inferiority or exploratory)	11-12
Changes to trial protocol	10	Important changes to the trial after it commenced including any outcomes or analyses that were not prespecified, with reason	Not applicable
Trial setting	11	Settings (such as community or hospital) and locations (eg, countries or sites) where the trial was conducted	11-12
Eligibility criteria	12a	Eligibility criteria for participants	11
	12b	If applicable, eligibility criteria for sites and for individuals delivering the interventions (eg, surgeons or physiotherapists)	Not applicable
Intervention and comparator	13	Intervention and comparator with sufficient details to allow replication. If relevant, where additional materials describing the intervention and comparator (eg, intervention manual) can be accessed	14-15
Outcomes	14	Prespecified primary and secondary outcomes, including the specific measurement variable (eg, systolic blood pressure), analysis metric (for example, change from baseline, final value, time to event), method of aggregation (eg, median, proportion), and time point for each outcome	12-14 & Supplementary Methods 2-4
Harms	15	How harms were defined and assessed (eg, systematically or non-systematically)	Figure 1
Sample size	16a	How sample size was determined, including all assumptions supporting the sample size calculation	15
	16b	Explanation of any interim analyses and stopping guidelines	Not applicable
Randomization:			
Sequence generation	17a	Who generated the random allocation sequence and the method used	12
	17b	Type of randomization and details of any restriction (eg, stratification, blocking and block size)	12
Allocation concealment mechanism	18	Mechanism used to implement the random allocation sequence (eg, central computer/telephone; sequentially numbered, opaque, sealed containers), describing any steps to conceal the sequence until interventions were assigned	12
Implementation	19	Whether the personnel who enrolled and those who assigned participants to the interventions had access to the random allocation sequence	12
Blinding	20a	Who was blinded after assignment to interventions (eg, participants, care providers, outcome assessors, data analysts)	12
	20b	If blinded, how blinding was achieved and description of the similarity of interventions	12
Statistical methods	21a	Statistical methods used to compare groups for primary and secondary outcomes, including harms	15-16
	21b	Definition of who is included in each analysis (eg, all randomized participants), and in which group	15-16 & Figure 1
	21c	How missing data were handled in the analysis	15-16
	21d	Methods for any additional analyses (eg. subgroup and sensitivity analyses), distinguishing prespecified from post hoc	Supplementary Methods 4-5
Participant flow, including flow diagram	22a	For each group, the numbers of participants who were randomly assigned, received intended intervention, and were analyzed for the primary outcome	Figure 1 & Table S1
	22b	For each group, losses and exclusions after randomization, together with reasons	Figure 1
Recruitment	23a	Dates defining the periods of recruitment and follow-up for outcomes of benefits and harms	11-12
	23b	If relevant, why the trial ended or was stopped	Not applicable
Intervention and comparator delivery	24a	Intervention and comparator as they were actually administered (eg, where appropriate, who delivered the intervention/comparator, how participants adhered, whether they were delivered as intended (fidelity))	14-15 & Supplementary Results 6-7
	24b	Concomitant care received during the trial for each group	14-15

Baseline data	25	A table showing baseline demographic and clinical characteristics for each group	30-31
Numbers analyzed, outcomes and estimation	26	For each primary and secondary outcome, by group:<br>• the number of participants included in the analysis<br>• the number of participants with available data at the outcome time point<br>• result for each group, and the estimated effect size and its precision (such as 95% confidence interval)<br>• for binary outcomes, presentation of both absolute and relative effect size	13 & 17-19 & 32-33 & Supplementary Results 6-7 & Table S1
Harms	27	All harms or unintended events in each group	Figure 1
Ancillary analyses	28	Any other analyses performed, including subgroup and sensitivity analyses, distinguishing pre-specified from post hoc	17-19 & Supplementary Results 6-7
Interpretation	29	Interpretation consistent with results, balancing benefits and harms, and considering other relevant evidence	20-24
Limitations	30	Trial limitations, addressing sources of potential bias, imprecision, generalisability, and, if relevant, multiplicity of analyses	22-23

Supplementary References

Author names in bold designate shared co-first authorship.

1. da Silva VS, Vieira MFS. International society for the advancement of kinanthropometry (Isak) global: International accreditation scheme of the competent anthropometrist. Revista Brasileira de Cineantropometria e Desempenho Humano. 2020;22:1-6. doi:10.1590/1980-0037.2020v22e70517

2. Martínez-González MA, García-Arellano A, Toledo E, et al. A 14-Item Mediterranean Diet Assessment Tool and Obesity Indexes among High-Risk Subjects: The PREDIMED Trial. PLoS One. 2012;7(8):e43134. doi:10.1371/JOURNAL.PONE.0043134

3. Dote-Montero M, Merchan-Ramirez E, Oses M, et al. Efficacy of different 8 h time-restricted eating schedules on visceral adipose tissue and cardiometabolic health: A study protocol. Nutr Metab Cardiovasc Dis. 2024;34(1):177-187. doi:10.1016/j.numecd.2023.09.014

4. Matthews DR, Hosker JP, Rudenski AS, et al. Homeostasis model assessment: insulin resistance and β-cell function from fasting plasma glucose and insulin concentrations in man. Diabetologia. 1985;28(7):412-419. doi:10.1007/BF00280883,

5. **Bolyen E, Rideout JR,** Dillon MR, et al. Reproducible, interactive, scalable and extensible microbiome data science using QIIME 2. Nature Biotechnology 2019 37:8. 2019;37(8):852-857. doi:10.1038/s41587-019-0209-9

6. Callahan BJ, McMurdie PJ, Rosen MJ, et al. DADA2: High-resolution sample inference from Illumina amplicon data. Nature Methods 2016 13:7. 2016;13(7):581-583. doi:10.1038/nmeth.3869

7. Glöckner FO, Yilmaz P, Quast C, et al. 25 years of serving the community with ribosomal RNA gene reference databases and tools. J Biotechnol. 2017;261:169-176. doi:10.1016/J.JBIOTEC.2017.06.1198

8. Douglas GM, Maffei VJ, Zaneveld JR, et al. PICRUSt2 for prediction of metagenome functions. Nature Biotechnology 2020 38:6. 2020;38(6):685-688. doi:10.1038/s41587-020-0548-6

9. Segata N, Izard J, Waldron L, et al. Metagenomic biomarker discovery and explanation. Genome Biol. 2011;12(6). doi:10.1186/GB-2011-12-6-R60

10. Nickols WA, Kuntz T, Shen J, et al. MaAsLin 3: Refining and extending generalized multivariable linear models for meta-omic association discovery. bioRxiv. Published online December 14, 2024. doi:10.1101/2024.12.13.628459

11. Benjaminit Y, Hochberg Y. Controlling the False Discovery Rate: A Practical and Powerful Approach to Multiple Testing. J R Stat Soc Series B Stat Methodol. 1995;57(1):289-300. doi:10.1111/J.2517-6161.1995.TB02031.X

12. Bakdash JZ, Marusich LR. Repeated Measures Correlation. Front Psychol. 2017;8(MAR):456. doi:10.3389/fpsyg.2017.00456

13. **Dote-Montero M, Clavero-Jimeno A,** Merchán-Ramírez E, et al. Effects of early, late and self-selected time-restricted eating on visceral adipose tissue and cardiometabolic health in participants with overweight or obesity: a randomized controlled trial. Nat Med. 2025;31(2):524-533. doi:10.1038/s41591-024-03375-y

14. Starekova J, Hernando D, Pickhardt PJ, et al. Quantification of Liver Fat Content with CT and MRI: State of the Art. Radiology. 2021;301(2):250-262. doi:10.1148/radiol.2021204288
