# Supplementary material for: Time-restricted eating, liver health, and fecal microbiota in adults with overweight or obesity: A randomized controlled trial
Source: JHEP Rep. 2026 Jul 7;8(11):101956. doi: 10.1016/j.jhepr.2026.101956 (PMC13661219; doi:10.1016/j.jhepr.2026.101956)
Supplement: Supplementary file 2 [file mmc2.docx]

**Journal of Hepatology**

**CTAT methods**

Tables for a “Complete, Transparent, Accurate and Timely account” (CTAT) are now mandatory for all revised submissions. The aim is to enhance the reproducibility of methods.

- Only include the parts relevant to your study
- Refer to the CTAT in the main text as ‘Supplementary CTAT Table’
- Do not add subheadings
- Add as many rows as needed to include all information
- Only include one item per row

**If the CTAT form is not relevant to your study, please outline the reasons why:**

1.1 **Antibodies**

| **Name** | **Citation** | **Supplier** | **Cat no.** | **Clone no.** |
| --- | --- | --- | --- | --- |
| N/A |  |  |  |  |

1.2 **Cell lines**

| **Name** | **Citation** | **Supplier** | **Cat no.** | **Passage no.** | **Authentication test method** |
| --- | --- | --- | --- | --- | --- |
| N/A |  |  |  |  |  |

1.3 **Organisms**

| **Name** | **Citation** | **Supplier** | **Strain** | **Sex** | **Age** | **Overall n number** |
| --- | --- | --- | --- | --- | --- | --- |
| N/A |  |  |  |  |  |  |

1.4 **Sequence based reagents**

| **Name** | **Sequence** | **Supplier** |
| --- | --- | --- |
| 341F | 5′TCGTCGGCAGCGTCAGATGTGTATAAGAGACAGCCTACGGGNGGCWGCAG | Illumina |
| 805R | 5′GTCTCGTGGGCTCGGAGATGTGTATAAGAGACAGGACTACHVGGGTATCTAATCC | Illumina |

1.5 **Biological samples**

| **Description** | **Source** | **Identifier** |
| --- | --- | --- |
| N/A |  |  |

1.6 **Deposited data**

| **Name of repository** | **Identifier** | **Link** |
| --- | --- | --- |
| NCBI Sequence Read Archive (SRA) | PRJNA1287213 | <https://dataview.ncbi.nlm.nih.gov/object/PRJNA1287213> |

1.7 **Software**

| **Software name** | **Manufacturer** | **Version** |
| --- | --- | --- |
| EasyDiet-Programa de Gestión de la Consulta® de la Academia Española de Nutrición y Dietética | Biocentury, S.L.U | March 2020 |
| R | R Foundation for Statistical Computing | v.4.4.2 |
| QIIME2 | Caporaso Lab | v.2024.10 |

1.8 **Other (e.g. drugs, proteins, vectors etc.)**

| N/A |  |  |
| --- | --- | --- |

1.9 **Please provide the details of the corresponding methods author for the manuscript:**

Jonatan R. Ruiz

Department of Physical Education and Sports, Faculty of Sport Sciences, University of Granada, Carretera de Alfacar s/n, 18071 Granada, Spain. Phone: +34 958-242-754. E-mail: [ruizj@ugr.es](mailto:ruizj@ugr.es)

**2.0	Please confirm for randomised controlled trials all versions of the clinical protocol are included in the submission. These will be published online as supplementary information.**

Yes
